# SerpinA3N attenuates ischemic stroke injury by reducing apoptosis and neuroinflammation

**DOI:** 10.1111/cns.13776

**Published:** 2021-12-12

**Authors:** Yu Zhang, Qianbo Chen, Dashuang Chen, Wenqi Zhao, Haowei Wang, Mei Yang, Zhenghua Xiang, Hongbin Yuan

**Affiliations:** ^1^ Department of Anesthesiology Second Affiliated Hospital of Naval Medical University Shanghai China; ^2^ Department of Anesthesiology Third Affiliated Hospital of Naval Medical University Shanghai China; ^3^ Department of Neurobiology Key Laboratory of Molecular Neurobiology Ministry of Education Naval Medical University Shanghai China

**Keywords:** AAV, Akt, clusterin, oxidative stress

## Abstract

**Objective:**

To assess the effect of serine protein inhibitor A3N (serpinA3N) in ischemic stroke and to explore its mechanism of action.

**Methods:**

Mouse ischemic stroke model was induced by transient middle cerebral artery occlusion followed by reperfusion. The expression pattern of serpinA3N was assessed using immunofluorescence, Western blot analysis, and real‐time quantitative PCR. An adeno‐associated virus (AAV) and recombinant serpinA3N were administered. Additionally, co‐immunoprecipitation‐mass spectrometry and immunofluorescence co‐staining were used to identify protein interactions.

**Results:**

SerpinA3N was upregulated in astrocytes and neurons within the ischemic penumbra after stroke in the acute phase. The expression of serpinA3N gradually increased 6 h after reperfusion, peaked on the day 2–3, and then decreased by day 7. Overexpression of serpinA3N by AAV significantly reduced the infarct size and improved motor function, associated with alleviated inflammation and oxidative stress. SerpinA3N treatment also reduced apoptosis both in vivo and in vitro. Co‐immunoprecipitation‐mass spectrometry and Western blotting revealed that clusterin interacts with serpinA3N, and Akt‐mTOR pathway members were upregulated by serpinA3N both in vivo and in vitro.

**Conclusions:**

SerpinA3N is expressed in astrocytes and penumbra neurons after stroke in mice. It reduces brain damage possibly via interacting with clusterin and inhibiting neuronal apoptosis and neuroinflammation.

## Graphical abstract

SerpinA3N‐overexpressing AAV was injected intracranially, and the overexpression effects were verified after 3 weeks by qRT‐PCR, Western blot, and immunofluorescence analysis. Neurological functions in mice 30 h after t‐MCAO were evaluated with Bederson's and Clark's systems. Overexpression of serpinA3N improves neurological function and reduces infarct volume after t‐MCAO.

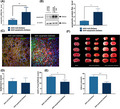

## 1 INTRODUCTION

Ischemic stroke is a major cause of death and disability worldwide. At present, the strategy for the treatment of acute ischemic stroke is to perform revascularization within a strict time window, leading to ischemic/reperfusion injury, which comes with a series of biochemical cascades.^1^ A previous study using an experimental ischemia model found that serine protein inhibitor A3N (serpinA3N) expression dramatically increases following stroke^1^, suggesting that it may play a role in stroke.

SerpinA3N, a murine orthologue of human α‐1‐antichymotrypsin, is a member of the serpin superfamily of protease inhibitors.^2^ Its folding is highly conserved, consisting of 8~9 α‐helices, 3 β‐sheets, and a solvent‐exposed stretch termed the reactive center loop (RCL), which interacts with the protease active site to promote protease activity.^3^, ^4^ A structure analysis revealed two features of serpinA3N: (1) the residues of the RCL are partially inserted into the A β‐sheet, a structure motif that is associated with ligand‐dependent activation in other serpins similar to non‐heparin‐activated antithrombin; and (2) two positively charged patches that might be associated with the binding of negatively charged entities such as DNA or glycosaminoglycans.^2^ Several proteases have been identified as substrates for serpinA3N, including antichymotrypsin, cathepsin G,^2^ leukocyte elastase,^2^, ^5^ granzyme B,^6^ and matrix metalloprotein 9 (MMP9).^7^ As an acute phase protein, serpinA3N is secreted in response to inflammation^7^, ^8^, ^9^, ^10^ and glucocorticoids^11^ as well as in various pathological conditions, such as an aortic aneurysm^12^ and colitis.^13^

In the central nervous system (CNS), serpinA3N is regarded as a potential marker of reactive astrogliosis.^1^, ^14^ Its function in the CNS is controversial. On the one hand, it induces neuroprotection, attenuates neuropathic pain,^5^ and reduces the severity of multiple sclerosis^6^ by inhibiting proteases. However, on the other hand, overexpression of serpinA3N in mouse hippocampus abolishes the protective effects of melatonin on trimethyltin chloride‐induced neuroinflammation and neurotoxicity.^15^ In ischemic stroke, its role remains unclear.

In the present study, we investigated serpinA3N expression levels and described its temporospatial distribution pattern in a mouse ischemic stroke model. We also evaluated whether it is neuroprotective against neuronal ischemic injury. Finally, the molecular mechanisms underlying its protective effects were explored.

## 2 MATERIALS AND METHODS

### 2.1 Animals

All experimental procedures were approved by the Institutional Animal Care and Use Committee at the Second Military Medical University. 8‐ to 12‐week‐old male C57BL/6 mice were purchased from Shanghai Jihui Laboratory Animal Care Co. Ltd. (Shanghai, China). Mice were housed on a 12‐h light/dark cycle with free access to food and water. All animals used in this work received care in compliance with the Guide for Care and Use of Laboratory Animals published by the National Institutes of Health, approved by the Animal Ethics Committee of the Second Military Medical University, and reported following the ARRIVE guidelines.^16^

### 2.2 Transient middle cerebral artery occlusion

A transient middle cerebral artery occlusion (MCAO) model was used to induce a focal cerebral ischemic stroke as previously described with modification.^17^ Briefly, mice were anesthetized with intraperitoneal injection of chloral hydrate (400 mg/kg). Cerebral focal ischemia was established by intraluminal occlusion of the right middle cerebral artery using a silicone rubber‐coated nylon monofilament (Guangzhou Jialing biotech Co., Ltd.), which was inserted and advanced through the carotid artery. Occlusion was verified by laser Doppler flowmetry (Moor Instruments, Inc.) with >70% reduction in regional blood flow perfusion. One hour after occlusion, the filament was withdrawn to allow for reperfusion and the general carotid artery was ligated. The skin was sutured, and the animal was allowed to recover. In sham‐operated mice, the same surgical procedure was performed, except that the monofilament was not inserted.

### 2.3 SerpinA3N overexpression with adeno‐associated virus

Adeno‐associated virus (AAV)‐serpinA3N‐ZsGreen was generated to overexpress serpinA3N carrying green fluorescence reporter Zs Green (Genomeditech Co., Ltd.), and AAV (AAV9,PGMAAV‐11994) carrying null‐ZsGreen served as controls. AAV was diluted in phosphate‐buffered saline (PBS) to 1 × 10^11^ genome copies/100 µl.

Four weeks prior to MCAO, mice were anesthetized and placed in a stereotaxic apparatus (Narishige). 2 μl of AAV suspension (2 × 10^9^ genome copies/mouse) was injected through a 36‐gauge glass cannula connected to a 2‐μl Hamilton syringe mounted on a microinjection pump (Univentor). The stereotaxic injection coordinates for the striatum were 2 mm posterior to bregma, 1.5 mm right of the midline, and 2.5 mm below the pia. The needle was kept in place for another 5 min before the cannula was slowly withdrawn to prevent reflux. The skin incision was then sutured, and the animal was kept warm with a heat blanket before being returned to the cage.

### 2.4 Neurologic functional scoring

Neurologic functions were evaluated 24 h after surgery by a researcher blinded to the groups using Bederson's Neurologic Examination Grading System^18^ (Table 1) and Clark's Focal Deficits Scoring System^19^ (Table 2). The tests described below were conducted sequentially.

**TABLE 1:** Bederson's neurologic examination grading system

Grade	Performance
0	No observable deficit
1	Forelimb flexion
2	Decreased resistance to lateral push (and forelimb flexion) without circling
3	Same behavior as grade 2, with circling

**TABLE 2:** Focal deficits (0–28)

	0	1	2	3	4
(1) Body symmetry (open bench top)	Normal	Slight asymmetry	Moderate asymmetry	Prominent asymmetry	Extreme asymmetry
(2) Gait (open bench top)	Normal	Stiff, inflexible	Limping	Trembling, drifting, falling	Does not walk
(3) Climbing (gripping surface, 45° angle)	Normal	Climbs with strain, limb weakness present	Holds onto slope, does not slip or climb	Slides down slope, unsuccessful effort to prevent fall	Slides immediately, no effort to prevent fall
(4) Circling behavior (open bench top)	Not present	Predominantly one‐sided turns	Circles to one side but not constantly	Circles constantly to one side	Pivoting, swaying, or no movement
(5) Front limb symmetry (mouse suspended by its tail)	Normal	Light asymmetry	Marked asymmetry	Prominent asymmetry	Slight asymmetry, no body/limb movement
(6) Compulsory circling (front limbs on bench, rear suspended by tail)	Not present	Tendency to turn to one side	Circles to one side	Pivots to one side sluggishly	Does not advance
(7) Whisker response (light touch from behind)	Symmetrical response	Light asymmetry	Prominent asymmetry	Absent response ipsilaterally, diminished contralaterally	Absent proprioceptive response bilaterally

### 2.5 Brain infarct volume

The mice were deeply anesthetized by chloral hydrate (600 mg/kg), and the brains were removed from the skull and were frozen at −20°C for 10 min. Frozen brains were then cut into five sections in the coronal plane and stained with 2,3,5‐triphenyltetrazolium chloride solution (TTC, Sigma‐Aldrich) at 37°C for 30 min before fixed in 4% formaldehyde for 10 min. The infarct areas were then measured, and infarct volumes were calculated using Image J software (NIH) by a laboratory assistant who was blinded to the study groups.

### 2.6 Mitochondria extraction

A mitochondria extraction kit (tissues) (Beyotime) was used to extract mitochondria according to the manufacturer's instructions. In brief, 80 mg of brain tissue was cut into small pieces and washed in PBS for 3 times. 640 µl mitochondria extraction buffer A was added, and tissue was ground for 20 s. After centrifugation at 600 g for 5 min, the supernatant was collected and centrifuged again at 11,000 g for 10 min. The supernatant containing cytoplasm was discarded, and pallet containing mitochondria was collected for further analysis.

### 2.7 Primary cell culture and oxygen‐glucose deprivation/reperfusion

CNS mixed glial cells were isolated from the cerebral cortices of postnatal (~24 h old) mice and cultured in Dulbecco's modified Eagle's medium (DMEM)/F12 containing 10% fetal bovine serum (D10; Gibco, Thermo Fisher) supplemented with 1% penicillin/streptomycin (Gibco). Cells were maintained at 37°C with 5% CO_2_ for 10 days until an astrocyte monolayer was formed.^20^ Primary microglia were shaken off at 180 rpm for 2h at 37°C and sub‐cultured in D10 media. Primary microglia were stimulated with interleukin‐4 (IL‐4, 10 ng/ml; Peprotech) or lipopolysaccharide (LPS, 1 µg/ml; Sigma‐Aldrich) in the presence or absence of recombinant serpinA3N (50 ng/ml; R&D Systems) for 24 h.

Cortical neurons were dissected from E18 mouse embryos. After digestion with trypsin, neuronal cells were suspended in high glucose DMEM (Gibco) containing 10% (v/v) equine serum and 25 μM L‐glutamine. Cells were seeded at a density of 5 × 10^4^/well in 6‐well tissue culture plates coated with 0.5 mg/ml poly‐L‐lysine (Gibco). After 24‐h incubation, the medium was replaced by Neurobasal medium with 2% B27 supplement (both from Gibco), 25 μM L‐glutamine, and 2.5 μg/ml cytosine arabinoside (Sigma‐Aldrich). Half of the media was replaced twice a week, and the cultures were used for experiments 7–8 days after plating.

For neuronal oxygen‐glucose deprivation/reoxygenation (OGD/R) model, medium was replaced by glucose‐free DMEM, and neurons were incubated in a hypoxic (0.1% O_2_) incubator for 4 h, as described previously.^17^ Neurons were then reoxygenated by returning to normal media and to the normoxic incubator (95% air/5% CO2) for 8 h. Recombinant serpinA3N (50 ng/ml) was added into the culture medium right before reoxygenation. Cells without any treatment served as the control group.

### 2.8 Cell viability assay

Neuron viability was determined using the Cell Counting Kit‐8 (CCK‐8, Dojindo) following the manufacturer's instructions. In brief, 50 μl of CCK‐8 reagent was added to each well for another 4 h at 37°C. Absorbance at 450 nm was measured using a microplate reader (BioTek).

### 2.9 Quantitative real‐time polymerase chain reaction

Quantitative real‐time polymerase chain reaction (qRT‐PCR) was performed as previously described.^17^ Total RNA was extracted from tissues or cultures using RNAfast200 kit (Fastagen Biotech) according to the manufacturer's instructions. PCR was then performed using a LightCycler 96 (Roche) and SYBRGreen PCR MIX (Takara) with the primers listed below. Gene expression levels were quantified using a cDNA standard curve, and data were normalized to the housekeeping gene β‐actin. Each reaction was performed in duplicate, and analysis was performed using the $2^{‐\Delta \Delta C_{t}}$ method. Data are expressed as fold changes.

SerpinA3N (Gene ID: 20716) primers:

**Table:** 

GGCTCTTGATGGCTGGGATC	F
TGTAGGAGGTGCCCAAAGCC	R

β‐actin (Gene ID: 11461) primers:

**Table:** 

GAGAAGCTGTGCTATGTTGCT	F
GTCTTTACGGATGTCAACGTCA	R

iNOS (Gene ID:18126) primers:

**Table:** 

GCCTCATGCCATTGAGTTCATC	F
TGTGCTGTGCTACAGTTCCGAG	R

COX‐2 (Gene ID:19225) primers:

**Table:** 

AGTCTTTGGTCTGGTGCCTG	F
TGGTAACCGCTCAGGTGTTG	R

Ncf1 (Gene ID:17969) primers:

**Table:** 

ATTCACCGAGATCTACGAGTTC	F
TGAAGTATTCAGTGAGAGTGCC	R

Ncf2 (Gene ID:17970) primers:

**Table:** 

GAAGATACCTCTCCAGAATCCG	F
TTCTTAGACACCATGTTCCGAA	R

TNFα (Gene ID:21926) primers:

**Table:** 

CAGGCGGTGCCTATGTCTC	F
CGATCACCCCGAAGTTCAGTAG	R

IL‐6 (Gene ID:16913) primers:

**Table:** 

TAGTCCTTCCTACCCCAATTTCC	F
TTGGTCCTTAGCCACTCCTTC	R

### 2.10 Immunohistochemistry

A modified immunofluorescence protocol was used based on previous reports.^21^, ^22^ Briefly, the mice were sacrificed and perfused through the aorta with a 0.9% NaCl solution. The brains were dissected, fixed in 4% paraformaldehyde, and then dehydrated with 25% sucrose. After rapidly freezing, floating slices were prepared by cutting in the coronal plane (20 μm in thickness, Leica cryostat). After washing with PBS, brain slices were incubated in antiserum solution (10% normal bovine serum, 0.2% Triton X‐100, 0.4% sodium azide in 0.01 mol/L PBS pH 7.2) for 30 min, followed by sequential incubation with primary antibodies (overnight at 4°C, Table 3) and Cy3 or FITC conjugated secondary antibodies (1:400, 2 h at room temperature, Jackson ImmunoResearch Labs). Images were taken with a Nikon digital camera DXM1200 (Nikon) attached to a Nikon Eclipse E600 microscope (Nikon) or with a confocal microscope (Zeiss LSM 710).

**TABLE 3:** Primary antibodies used in this study

Primary antibodies	Host	Company	Dilution		
			IF	WB	Co‐IP
SerpinA3N	Goat	R&D	1:300	1:5000	1:50
NeuN	Mouse	Millipore	1:100		
GFAP	Mouse	Boster	1:100		
Iba1	Rabbit	Abcam	1:1000		
S100b	Mouse	Boster	1:100		
Clusterin‐α	Mouse	Santa Cruz	1:100	1:500	1:50
GFP	Mouse	Santa Cruz	1:200		
Bcl2	Rabbit	Beyotime		1:1000	
bax	Rabbit	Proteintech		1:1000	
Caspase‐3	Rabbit	CST		1:2000	
Cleaved caspase‐3	Rabbit	CST		1:500	
iNOS	Rabbit	Boster		1:1000	
TNFα	Mouse	Boster		1:200	
p‐p38	Rabbit	CST		1:1000	
nNOS	Rabbit	Boster		1:1000	
Akt	Rabbit	CST		1:2000	
p‐Akt	Rabbit	CST		1:2000	
mTOR	Rabbit	CST		1:1000	
p‐mTOR	Rabbit	CST		1:1000	
pCREB	Rabbit	Santa Cruz		1:500	
β‐actin	Mouse	Beyotime		1:10000	
β‐tubulin	Mouse	Beyotime		1:5000	

In this study, neurons, reactive astrocytes, macrophage/microglia, and oligodendrocyte lineage cells were, respectively, indicated by NeuN^+^,^23^ S100B^+^,^24^ iba1^+^,^25^ and olig2^+^ cells.^26^ SerpinA3N^+^ cells and double‐positive cells were manually counted by two laboratory assistants who were blinded to the study groups.

### 2.11 Western blot and co‐immunoprecipitation

Tissue lysates were prepared with RIPA buffer (30 mM HEPES [PH 8.0], 150 mM NaCl, 1% NP‐40, 10 mM NaF, 1 mM EDTA) containing protease inhibitors cocktail (Bytotime), and insoluble debris was removed by centrifugation at ‐12,000 g for 10 min at 4°C. The protein concentration of the supernatant was determined using the Bradford method.

Western blotting was performed as described previously.^17^ Protein fractions were separated by 10% or 12% SDS‐PAGE and transferred onto nitrocellulose membranes. After blocking with 5% bovine serum albumin in 0.1% (v/v) Tween‐20 in tris‐buffered saline (TBS), membranes were sequentially incubated with primary antibodies (Table 3) and HRP‐conjugated secondary antibodies. Protein bands were developed with enhanced chemiluminescence (ECL) substrate solution (Beyotime) and visualized using a BIO‐RAD Molecular Imager (Bio‐Rad laboratories Inc).

Co‐immunoprecipitation (Co‐IP) was performed as previously described.^27^ Briefly, 300–500 μl of tissue lysates was incubated with 0.5–2 μg of the corresponding antibodies (Table 3) for 3 h at 4 °C. 50 μl of Protein G‐agarose beads (Beyotime) was then added and incubated overnight. After washing, samples were boiled for 3–5 min in sample‐loading buffer, then subjected to SDS‐PAGE and Western blotting as described above.

### 2.12 Liquid chromatography‐tandem mass spectrometry

The proteins pulled‐down by IP were subjected to liquid chromatography‐tandem mass spectrometry (LC‐MS‐MS) analysis performed by Omics Space, Shanghai, China. Shotgun proteomics were used allowing for powerful separation of liquid chromatography in combination with highly sensitive and selective mass analysis.

### 2.13 Statistical analysis

Statistical analyses were performed with Prism 8 (GraphPad). Normality was tested using Shapiro‐Wilk test. Data of normal distribution are expressed as the mean ± SEM and evaluated using an unpaired *t* test or ANOVA followed by the Tukey's post hoc test. Data of non‐normal distribution are expressed as median [quartile] and evaluated using Mann‐Whitney *U* test. Significance was set at *p* < 0.05.

## 3 RESULTS

### 3.1 Temporospatial distribution patterns of serpinA3N after stroke

**FIGURE 1:**
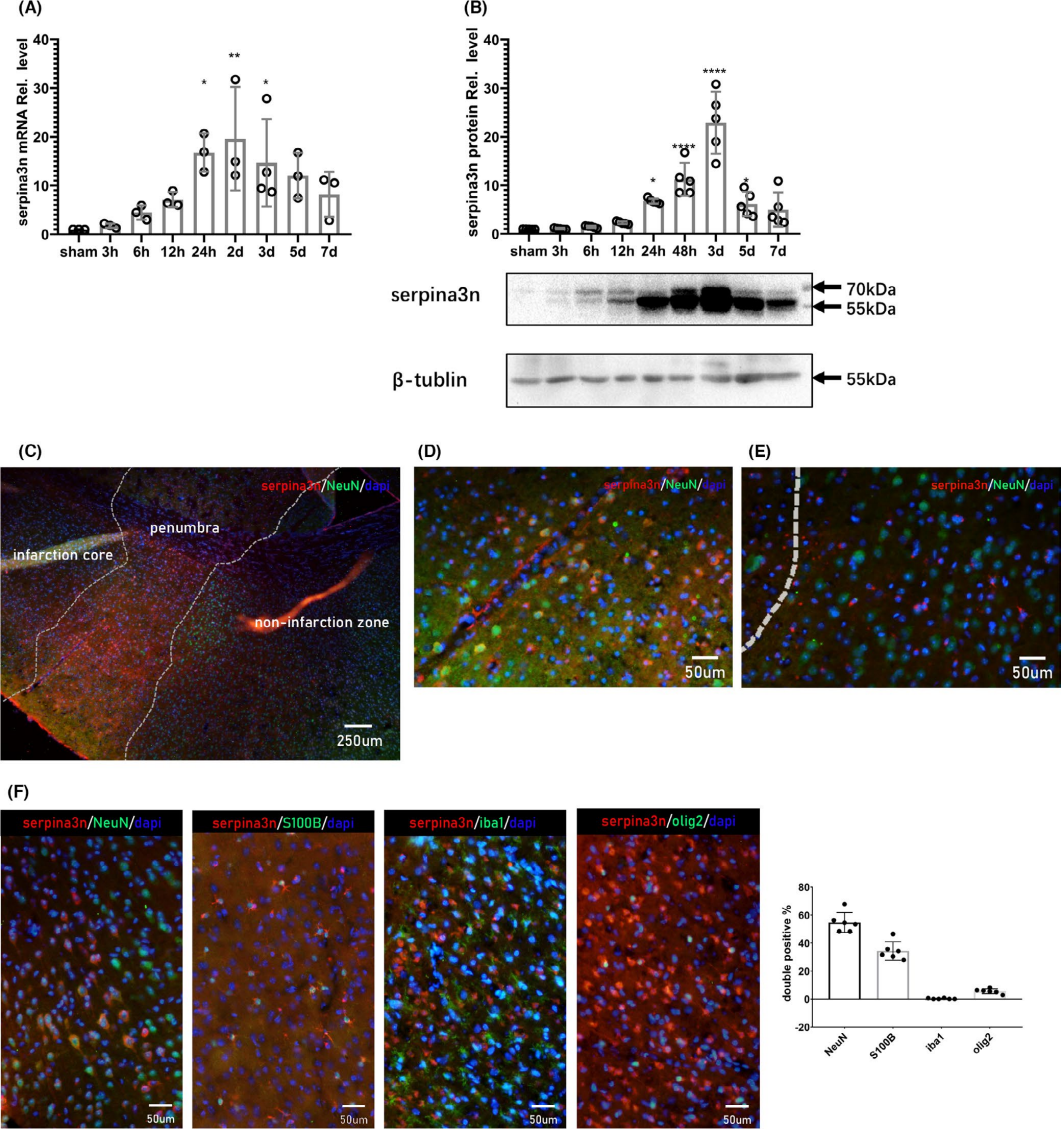
Temporospatial distribution patterns of serpinA3N after MCAO. (A) qPCR assay and (B) Western blot analysis of serpinA3N expression at indicated time points after MCAO in mice. *N* = 3. *, **, ***, and **** *p* < 0.05, 0.01, 0.001, and 0.0001. (C–F) Immunostaining of serpinA3N with cell markers (C–E) NeuN, (F) S100b, Iba1, and Olig2 24 h after t‐MCAO, with percentage of double positive cells in total serpinA3N+ cells. Scale bar = 250 μm (C) and 50 μm (D–F)

To examine the temporal expression pattern of serpinA3N after stroke, serpinA3N mRNA and protein expression levels were detected at different time points. We found that serpinA3N mRNA expression increased 24 h after reperfusion, peaked at 2 days, and then gradually declined (Figure 1A). SerpinA3N protein expression showed a similar pattern, which increased at 24 h, peaked at 3 days, a bit delayed compared to mRNA levels, and then gradually decreased (Figure 1B).

The spatial distribution pattern of serpinA3N was examined by immunofluorescence analysis 24 h after reperfusion. Infarct core, penumbra, and non‐infarct zone were identified depending on the intensity of NeuN signals and the morphology of Iba1^+^ microglia (Figure 1C & Figure S1A). Increased serpinA3N expression was observed in the penumbra and non‐infarction zone (Figure 1C). In the penumbra, most of the serpinA3N expressing cells were NeuN^+^ (Figure 1D). Interestingly in the non‐infarction zone, serpinA3N expressing cells were NeuN^−^ and significantly smaller in size (Figure 1E). Further analysis showed those smaller serpinA3N^+^ cells were actually S100b^+^ (Figure 1F), indicating reactive astrocytes.^24^

### 3.2 Overexpression of serpinA3N improves neurologic function and reduces infarct volume following stroke

SerpinA3N‐overexpressing AAV was injected intracranially into the striatum. Three weeks after, AAV was widely distributed in the striatum (Figure S1A), predominantly in the penumbra area (Figure S1B). As for the cellular distribution, we observed significantly colocalization of ZsGreen with NeuN (Figure S1C), suggesting neuronal infection of the AAV.

**FIGURE 2:**
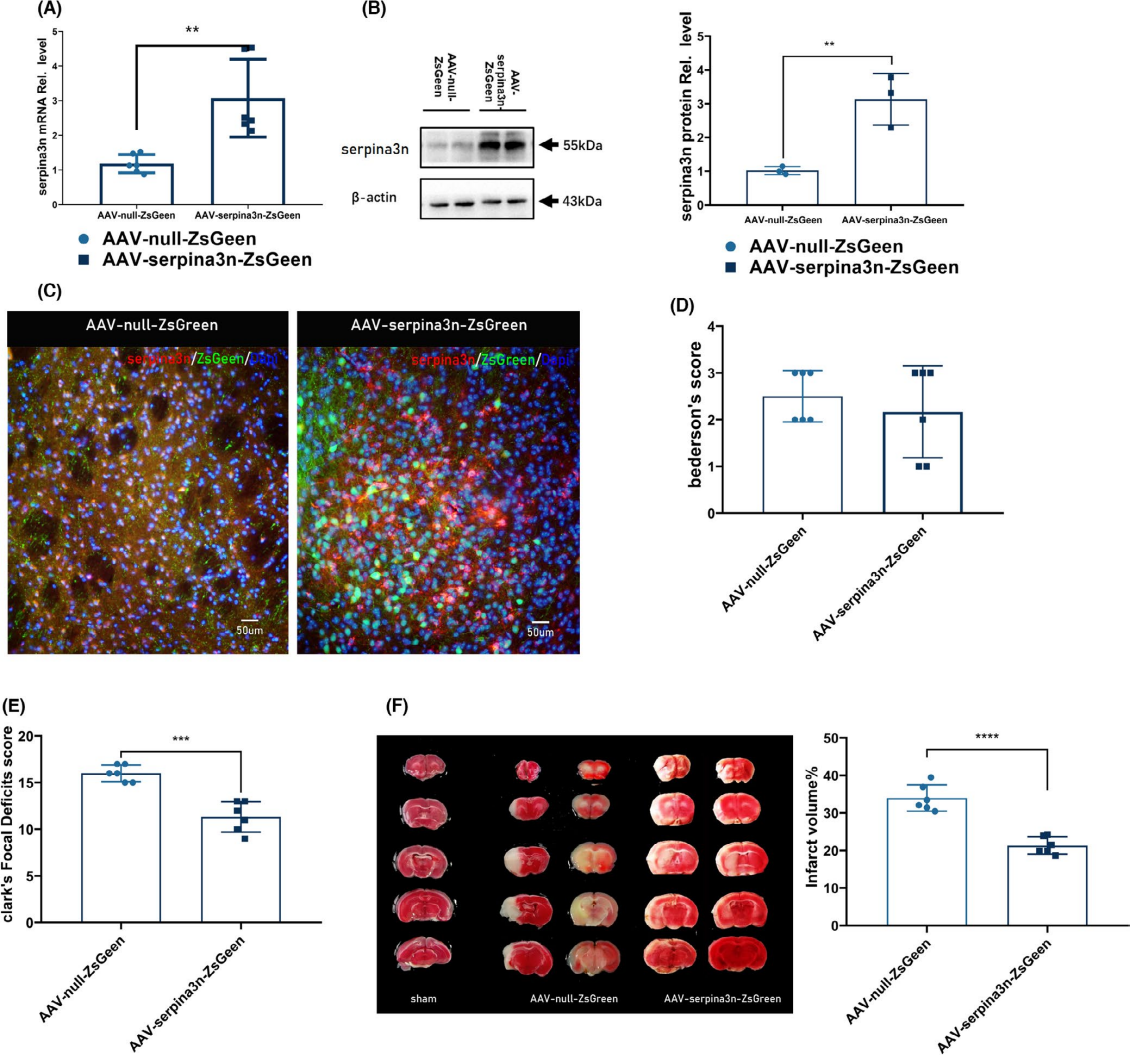
Overexpression of serpinA3N improves neurologic function and reduces infarct volume after MCAO. (A) qPCR assay and (B) Western blot analysis serpinA3N expression following MCAO 3 weeks after AAV injection. (C) Immunofluorescence of serpinA3N and ZsGeen 24 h after MCAO. (D) Bederson's Neurologic Examination Grading System and (E) Clark's Focal Deficits Scoring System on neurologic evaluation 30 h after t‐MCAO. (F) Brain infarct volume and quantification assessed by TTC staining. *N* = 6. ** and ****p < 0.01 and 0.0001

Overexpression effects were verified 3 weeks after AAV injection by qRT‐PCR (Figure 2A), Western blot (Figure 2B), and immunostaining (Figure 2C). Neurologic functions in mice 30 h after MCAO were evaluated with Bederson's and Clark's systems, while no difference was found in the Bederson's system (Figure 2D). SerpinA3N overexpression resulted in lower scores in the Clask's system (Figure 2E). In accordance with the neurologic tests, serpinA3N overexpression significantly reduced infarct volumes (Figure 2F).

### 3.3 SerpinA3N inhibits the pro‐inflammatory and oxidative responses after stroke

**FIGURE 3:**
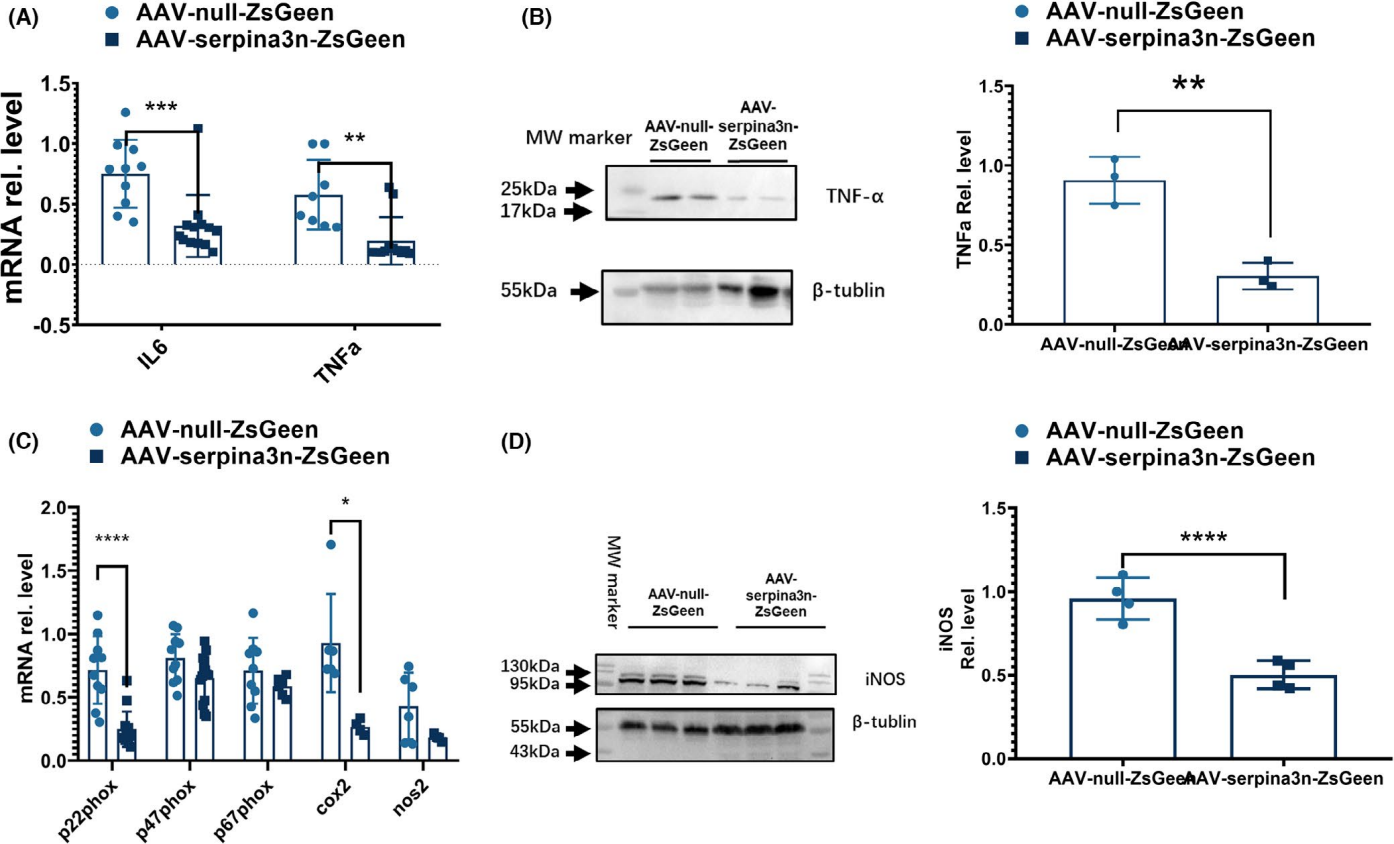
SerpinA3N inhibits the pro‐inflammatory and oxidative responses after stroke. Mice were injected with AAV‐null‐ZsGreen or AAV‐serpinA3N‐ZsGreen, and brain tissue lysates were harvested 30 h after MCAO. (A) qPCR assay on IL‐6 and TNFα mRNA expression. (B) Western blot analysis of TNFα expression. (C) qPCR assay on the expressions of p22phox, p47phox, p67phox, COX‐2, and iNOS. (D) Western blot analysis of iNOS expression. *, **, ***, and **** *p* < 0.05, 0.01, 0.001, and 0.0001

To evaluate the effects of serpinA3N on pro‐inflammatory responses after stroke, we measured the expression of inflammatory cytokines interleukin (IL)‐6 and tumor necrosis factor (TNF)‐α and found that IL‐6 and TNF‐α were significantly lower in serpinA3N‐overexpressed mice at 24 h poststroke (Figure 3A,B). We also evaluated the oxidative stress molecules including p22^phox^, p47^phox^, and p67^phox^, critical components for superoxide generation through the NAPDH oxidase system, and cyclooxygenase (cox)‐2 and nitric oxide synthase 2 (encoding inducible nitric oxide synthase [iNOS]), two critical enzymes in the synthesis of reactive oxygen species and nitric oxide. We found that the mRNA expression of p22^phox^ subunit and cox2 (Figure 3C) and the protein level of iNOS (Figure 3D) were significantly decreased by serpinA3N overexpression.

Microglia are brain resident immune cells and are a critical source of neuroinflammation and oxidative stress.^28^, ^29^, ^30^ To determine whether serpinA3N directly affects microglial phenotype, primary microglial cultures were treated with LPS to induce M1 polarization in the presence of serpinA3N. We found that serpinA3N failed to alter M1 and M2 markers^31^, ^32^, ^33^ (Figure S2) after LPS stimulation. These results indicate that serpinA3N reduces inflammation and oxidative stress indirectly, rather than directly activating microglia or changing their polarizing status.

### 3.4 SerpinA3N decreases apoptosis both in vitro and in vivo

**FIGURE 4:**
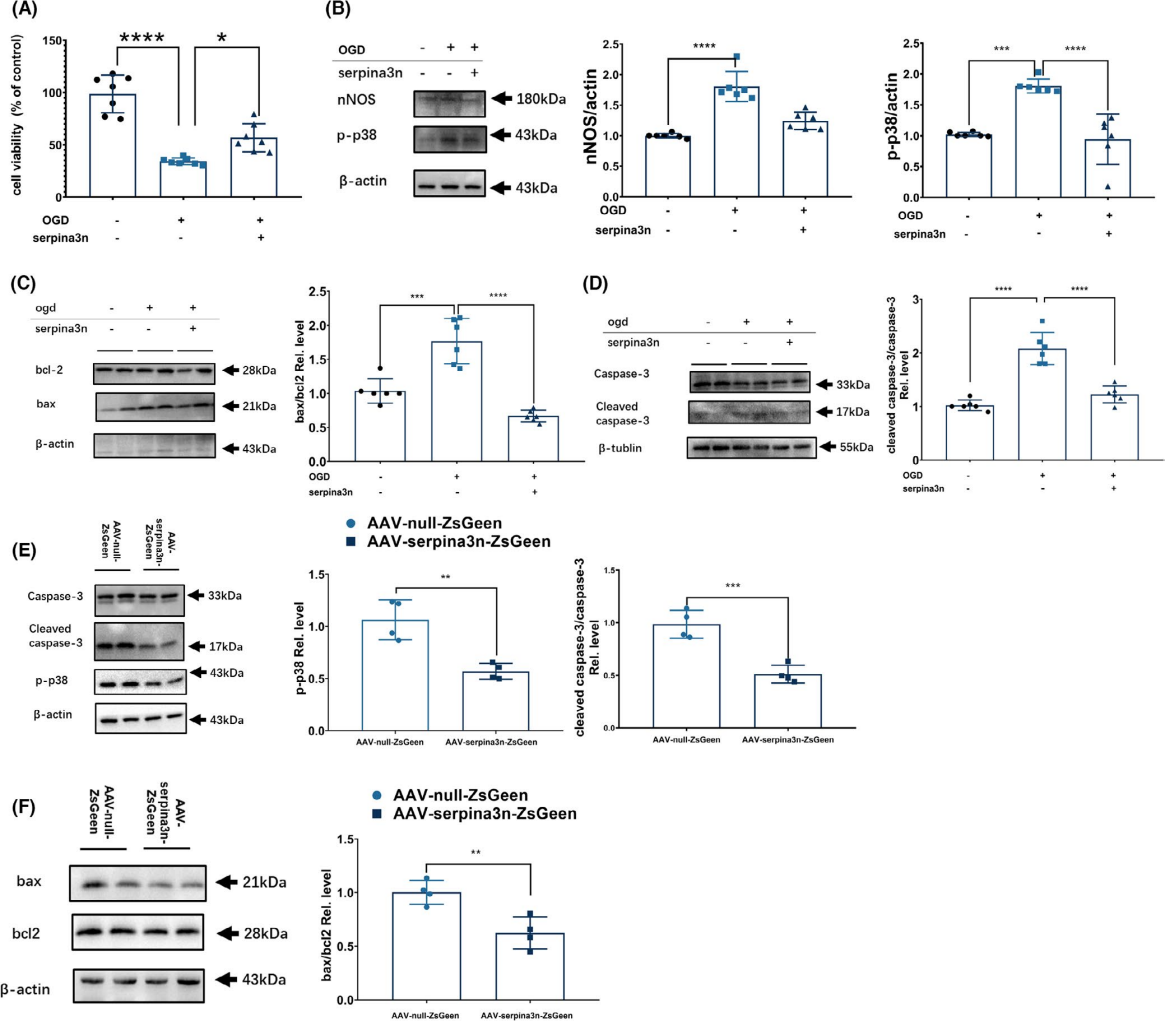
SerpinA3N decreases apoptosis both in vitro and in vivo. Primary neuronal cultures were subjected to OGD/R, with or without serpinA3N treatment. Analyses were performed 4 h after OGD/R. (A) Cell viability evaluated by CCK‐8 kit. (B) Western blot analysis of nNOS and phosphorylated p38 levels (C–D) Western blot analysis of apoptosis‐related proteins Bcl‐2, Bax, Caspase‐3, and cleaved Caspase‐3. *N* = 6–7. *, ***, and **** *p* < 0.05, 0.001, and 0.0001. Mice were injected with AAV‐null‐ZsGreen or AAV‐serpinA3N‐ZsGreen, and brain tissue lysates were harvested 30 h after t‐MCAO. Western blot analysis of protein expressions of (E) phosphorylated p38, Caspase‐3, and cleaved Caspase‐3 (F) Bax and Bcl‐2. *N* = 4. ** and ***, *p* < 0.01 and 0.001

Apoptosis serves as a major mechanism responsible for neuronal loss after ischemic stroke. We therefore detected the effect of serpinA3N on apoptosis both in vitro and in vivo. In primary neuronal cultures, a dose‐response experiment of recombinant serpinA3N was performed and founded that serpinA3N treatment promoted cell survival measured 4 hours after OGD/R with the dose of 50 ng/ml (Figure 4A, Figure S3). Both phospho‐p38 (p‐p38) and neuronal nitric oxide synthase (nNOS) are associated with neuronal apoptosis,^34^, ^35^ and both were found to be downregulated by serpinA3N (Figure 4B). As expected, apoptosis indicators including the ratios of Bax/Bcl‐2 and cleaved Caspase‐3/Caspase‐3 were both brought down by serpinA3N, proving serpinA3N an effective antiapoptotic agent. Similar findings were also present in stroke mice in vivo, where overexpression of serpinA3N reduced p‐p38, Bax/Bcl‐2, and cleaved Caspase‐3/Caspase‐3 (Figure 4C–F).

### 3.5 Identification of clusterin as a SerpinA3N‐interacting protein

We then try to identify the molecular mechanisms of serpinA3N’s protective effects by detecting serpinA3N‐interacting proteins. SerpinA3N was pulled‐down from mouse brains 24 h poststroke (*n* = 3 for each time point) by IP using an anti‐serpinA3N antibody or IgG control, with agarose beads conjugated to protein A+G. Proteins that were pulled‐down underwent LC‐MS‐MS analysis, and results from each brain are shown in Table 4. A total of 47 unique proteins were identified, among which 3 of them (SerpinA3K, Clusterin, and Rps25) were significantly different (1.5 folds and *p* < 0.05) between the two groups (Figure S4). Combining what we found with the data from the interaction network of serpinA3N (http://www.string‐db.org/, Figure S4), we focused on clusterin, a member of small heat shock protein family and protein chaperone associated with apoptosis.^36^

**TABLE 4:** Basic information of LCMS/MS results

Sample	Protein	Protein (unique peptide ≥1)	Peptide
SerpinA3N No.1	1277	1182	6744
SerpinA3N No.2	1167	1048	6100
SerpinA3N No.3	924	761	3645
IgG No.1	1099	956	4734
IgG No.2	1021	881	4990
IgG No.3	1096	982	5882

Co‐IP/MS results indicated that most of the serpinA3N‐interacting proteins are associated with protein synthesis and oxidation respiratory chain enzymes, which are located in mitochondria. Western blotting with brain tissue lysates proved the presence of serpinA3N (Figure S5A), which was predominantly in the cytosol while barely detectible in the mitochondria (Figure S5B).

**FIGURE 5:**
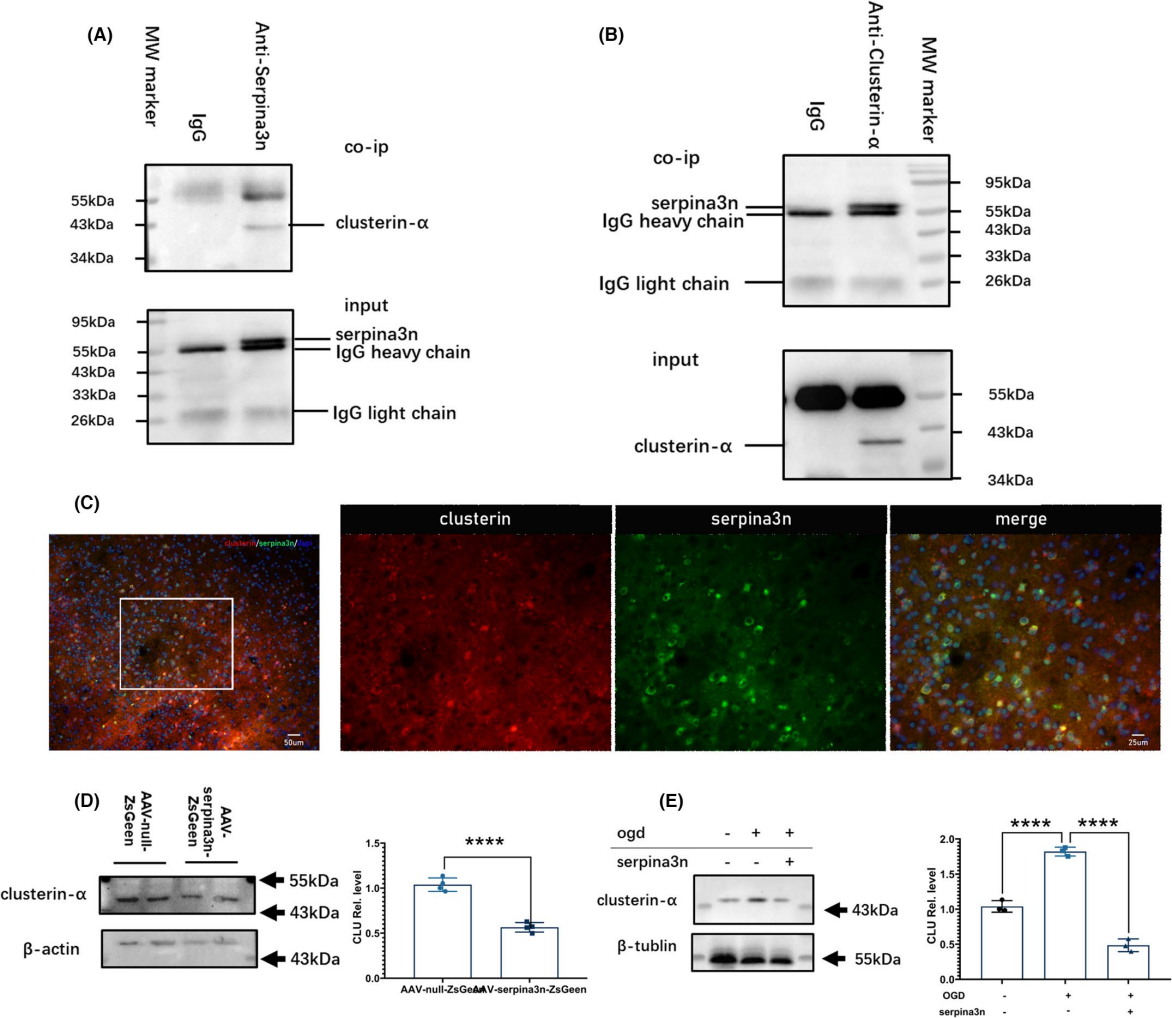
Identification of clusterin as a SerpinA3N‐interacting protein. (A) Co‐immunoprecipitation of clusterin‐α using an anti‐serpinA3N antibody. (B) Co‐immunoprecipitation of serpinA3N using an anti‐clusterin‐α antibody. (C) Immunostaining of serpinA3N and clusterin‐α in mouse brain 24 h after MCAO. (D) Mice were injected with AAV‐null‐ZsGreen or AAV‐serpinA3N‐ZsGreen, and clusterin‐α was detected with Western blotting. *N* = 4. ****p < 0.0001. (E) Primary neuronal cultures were subjected to OGD/R with or without serpinA3N treatment, and clusterin‐α was detected with Western blotting. *N* = 4. ****p < 0.0001

To further confirm the interaction between serpinA3N and clusterin, we performed a Co‐IP assay. The brain lysates were IP‐ed by anti‐clusterin α subunit (clusterin‐α), and serpinA3N was blotted (Figure 5A), and then, IP‐ed by anti‐serpinA3N with clusterin‐α blotted (Figure 5B). Both consistently confirmed the binding of serpinA3N with clusterin‐α. As expected, immunofluorescence double‐staining results confirmed that serpinA3N and clusterin‐α are colocalized in cortical neurons after stroke (Figure 5C).

We then detected the effect of serpinA3N on clusterin‐α expression. Western blot analysis showed that serpinA3N overexpression reduced clusterin‐α in mouse brain 30h after MCAO (Figure 5D), and in cultured neuron after OGD/R (Figure 5E). These findings suggest that serpinA3N reduced clusterin‐α, probably through their interaction.

### 3.6 SerpinA3N increases activation of Akt signaling pathway

**FIGURE 6:**
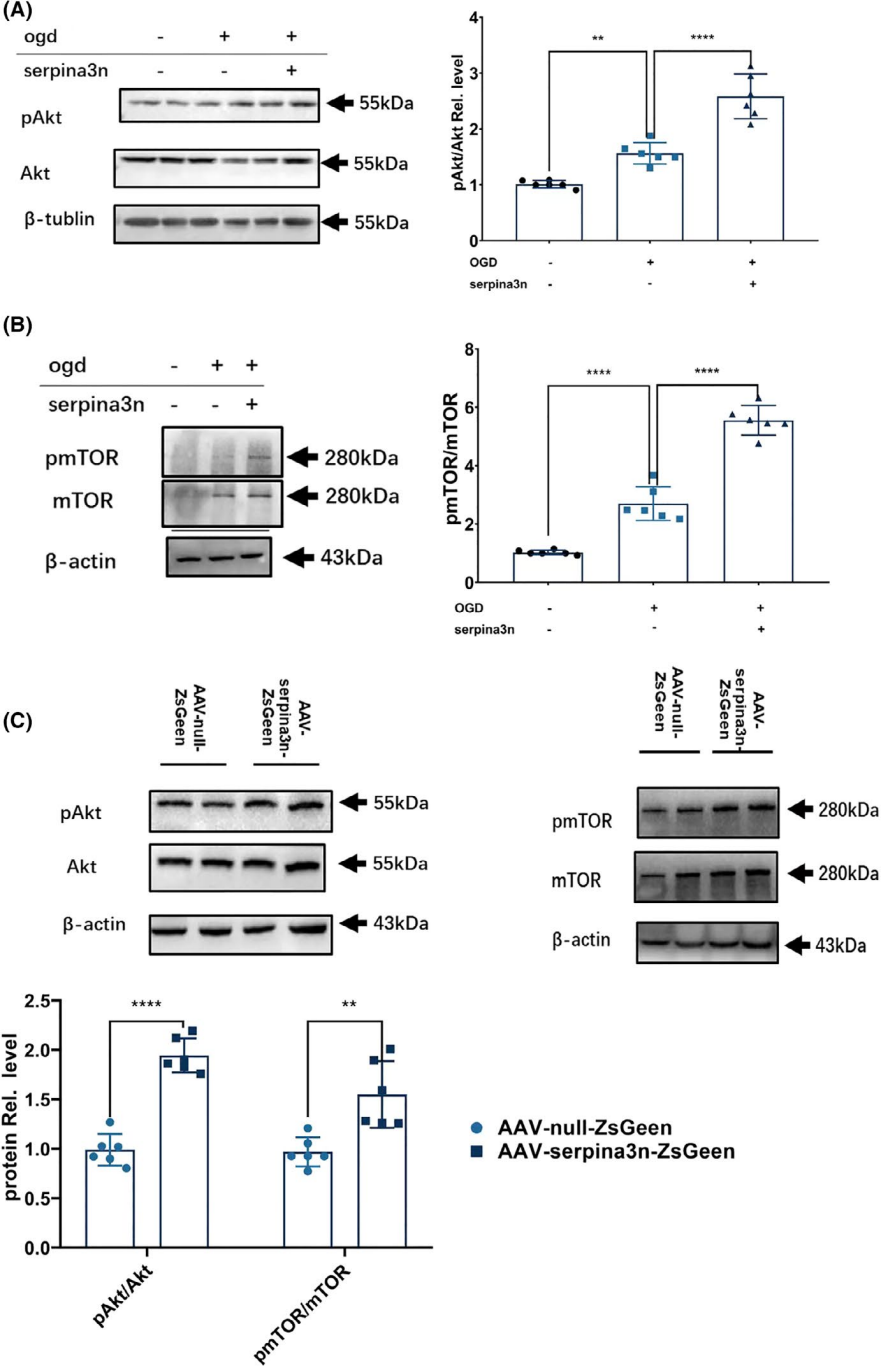
SerpinA3N increases the activation of Akt signaling pathway. Primary neuronal cultures were subjected to OGD/R with or without serpinA3N treatment. Western blot analysis of (A) Akt phosphorylation and (B) mTOR phosphorylation 4 h after OGD/R. *N* = 6. ** and ****p < 0.01 and 0.0001. Mice were injected with AAV‐null‐ZsGreen or AAV‐serpinA3N‐ZsGreen. (C) Western blot analysis of Akt phosphorylation and mTOR phosphorylation 30 h after t‐MCAO. *N* = 6. ** and ****p < 0.01 and 0.0001

Previous reports demonstrated that clusterin enhances cell survival via activating the PI3K/Akt pathways in retinal glial cells and in other peripheral tissues such as the pulmonary artery, the liver, and the sperm cells.^37^, ^38^, ^39^, ^40^ To explore whether this serves as a potential mechanism for serpinA3N‐afforded neuronal cell survival, we detected the Akt activation and its downstream protein, mechanistic target of rapamycin (mTOR), both in vitro and in vivo. In neuronal cultures subjected to OGD/R, serpinA3N treatment increased both Akt activation and mTOR activation, indicated by the ratios of phospho‐Akt (p‐Akt)/Akt and phospho‐mTOR (p‐mTOR)/mTOR, respectively (Figure 6A‐B). Similar findings were also observed in mouse brain lysates after MCAO (Figure 6C). These results suggest that serpinA3N protects the neurons against apoptosis associated with activation of the Akt signaling pathway.

## 4 DISCUSSION

In the present study, we confirmed the upregulation of serpinA3N after ischemic stroke and described its temporospatial distribution poststroke. We then reported that overexpression of serpinA3N in vivo elicited neuroprotection associated with suppressed neuroinflammation, oxidative stress, and neuronal apoptosis. Finally, we proved the molecular interaction between serpinA3N and clusterin, a molecular chaperone, as well as the participation of Akt‐mTOR pathway, which may serve as the underlying mechanism for serpinA3N’s antiapoptotic effects.

Consistent with previous study^1^, we found that serpinA3N expression was upregulated at 6–12 h after reperfusion and lasted for 3 days. Notably, although the serpinA3N expression levels began to drop after 3 d, they remained significantly higher even 7 d after MCAO compared baseline. SerpinA3N was mainly expressed in neurons and astrocytes, with a few serpinA3N‐positive cells being oligodendrocyte lineage cells, and no serpinA3N expression in microglia. This was controversial with the previous belief that serpinA3N was a marker of reactive astrocytes^1^. Although most reported the expression of serpinA3N in reactive astrocytes in various models,^14^, ^15^, ^41^ two recent studies revealed serpinA3N expression in neurons in schizophrenia model^42^ and in oligodendrocytes in Alzheimer's disease (AD).^43^ We are the first to report serpinA3N cellular distribution in stroke model.

Clusterin is traditionally referred to as an extracellular chaperone. It is composed of two 35–40 kD subunits (α and β) encoded by a single gene and held together by disulfide bonds. Clusterin has a variety of functions, including regulation of apoptosis, protecting cells at fluid‐tissue interfaces from stress, transporting lipids, and regulating complement activity.^44^ In CNS, clusterin mRNA is detected in neurons and glia, and its expression is upregulated in astrocytes in a variety of disease states including AD, stroke, and seizure.^44^ Its role in CNS ischemia/hypoxia is controversial. On the one hand, clusterin contributed to caspase‐3‐independent brain injury following neonatal hypoxia‐ischemia^44^ and caused a substantial neuronal loss and synaptic dysfunction in organotypic hippocampal slice cultures subjected to OGD.^45^ In addition, clusterin activated microglia and promoted the release of nitrite oxide and TNF‐α in vivo and in vitro.^46^ However, on the other hand, clusterin was observed to be neuroprotective in focal cerebral ischemia, as evidenced by thinner size of the penumbra, less apoptotic cells at day 7 after ischemia,^47^ and improved remodeling in late ischemic phase.^48^

In the present study, serpinA3N was barely detectable in mitochondrial extracts from whole brain tissue. This is in concert with previous reports, which showed that serpinA3N was a secreted protein. In our study, direct serpinA3N‐clusterin interactions were verified, and serpinA3N overexpression reduced the clusterin level after ischemic insults in vivo and in vitro, suggesting that serpinA3N might influence clusterin synthesis or degradation, which needs further investigation.

Stroke triggers an inflammatory response, particularly in microglia, induced by necrotic cells and debris and reactive oxygen species among others. These triggering factors promote astrocyte activation,^49^ lead to cytokine production, and induce adhesion molecule expression within the cerebral vasculature, all within 24 h of the ischemic insult.^50^ In our study, we demonstrated that serpinA3N overexpression alleviates MCAO‐induced inflammation, without direct influence on primary microglia activation or LPS‐induced microglia polarization in vitro (Figure S1). Based on the fact that serpinA3N interacts with clusterin and that clusterin can activate microglia in vivo and in vitro,^46^ the anti‐inflammation effect of serpinA3N might be due to the its inhibitory effects on clusterin.

In the present study, we also reported that serpinA3N decreases neuronal apoptosis. Previous studies have identified serpinA3N as an inhibitor of serine protease granzyme B and leukocyte elastase,^5^, ^6^ both could cause apoptosis.^51^, ^52^, ^53^ Although our co‐IP/MS results were unable to identify any serine proteases (data not shown), it led us to consider clusterin produced by cultured neurons, instead of microglia.^54^ Previous study shows the deleterious effect of clusterin to neurons related to N‐methyl‐d‐aspartic acid (NMDA) receptors.^45^ Another possibility is through inhibition of a pro‐apoptotic neuronal membrane macromolecular complex, composed of postsynaptic density protein‐95 (PSD‐95), NMDA receptors (NMDAR), nNOS, and scavenger receptors including low‐density lipoprotein receptor related protein (LRP)‐1, LRP‐2, very low‐density protein receptor, and apolipoprotein E receptor 2.^55^, ^56^, ^57^, ^58^, ^59^ Interestingly, LRP1 is critical for NMDAR surface distribution and internalization^60^ and NMDA‐induced PSD‐95 degradation.^61^ Our result that serpinA3N treatment reduced nNOS and p‐p38^62^, ^63^ might be suggestive of an inhibitory role for clusterin in NMDAR‐related neuropathies.

Previous reports demonstrated that clusterin enhances neuronal survival via activating the PI3K/Akt and other related signaling pathways.^37^, ^38^, ^39^, ^64^ Accordingly, we found that serpinA3N enhanced the activation of the Akt pathway in vivo and in vitro, which is associated with a neuroprotective effect.^65^, ^66^, ^67^, ^68^

A drawback of the present study is that we did not perform serpinA3N knockout or knockdown, given that serpinA3N itself is upregulated post‐MCAO. Nevertheless, we have proved that additional upregulation of serpinA3N expression elicited beneficial effects against stroke, which has some translational value. Further study should use knockout or knockdown approaches to prove the indispensable role of serpinA3N in neuroprotection. Another drawback is that only male animals were included in the present study. Sex dimorphism is well‐known in microglial inflammatory response.^40^, ^69^ Therefore, the results from this study may not be able to be generalized into female individuals.

## 5 CONCLUSIONS

SerpinA3N is expressed in astrocytes and penumbral neurons after stroke in mice and reduces damage possibly via interacting with clusterin and inhibiting neuronal apoptosis.

## CONFLICT OF INTEREST

The authors declare that they have no conflict of interest.

## AUTHOR CONTRIBUTIONS

Yu Zhang made equal contribution in roles/writing—original draft and methodology. Qianbo Chen involved in roles/writing—original draft and data curation. Dashuang Chen involved in formal analysis. Wenqi Zhao involved in data curation. Haowei Wang designed the methodology. Mei Yang involved in data curation and formal analysis. Zhenghua Xiang conceptualized the study and designed the methodology. Hongbin Yuan involved in conceptualization and writing—review and editing.

## Supporting information

Fig S1

Fig S2

Fig S3

Fig S4

Fig S5

Supplementary Material

## Data Availability

The data that support the findings of this study are available from the corresponding author upon reasonable request.
