# Supplementary figures and images for: SerpinA3N attenuates ischemic stroke injury by reducing apoptosis and neuroinflammation
Source: CNS Neurosci Ther. 2021 Dec 12;28(4):566–79. doi: 10.1111/cns.13776 (PMC8928918; doi:10.1111/cns.13776)

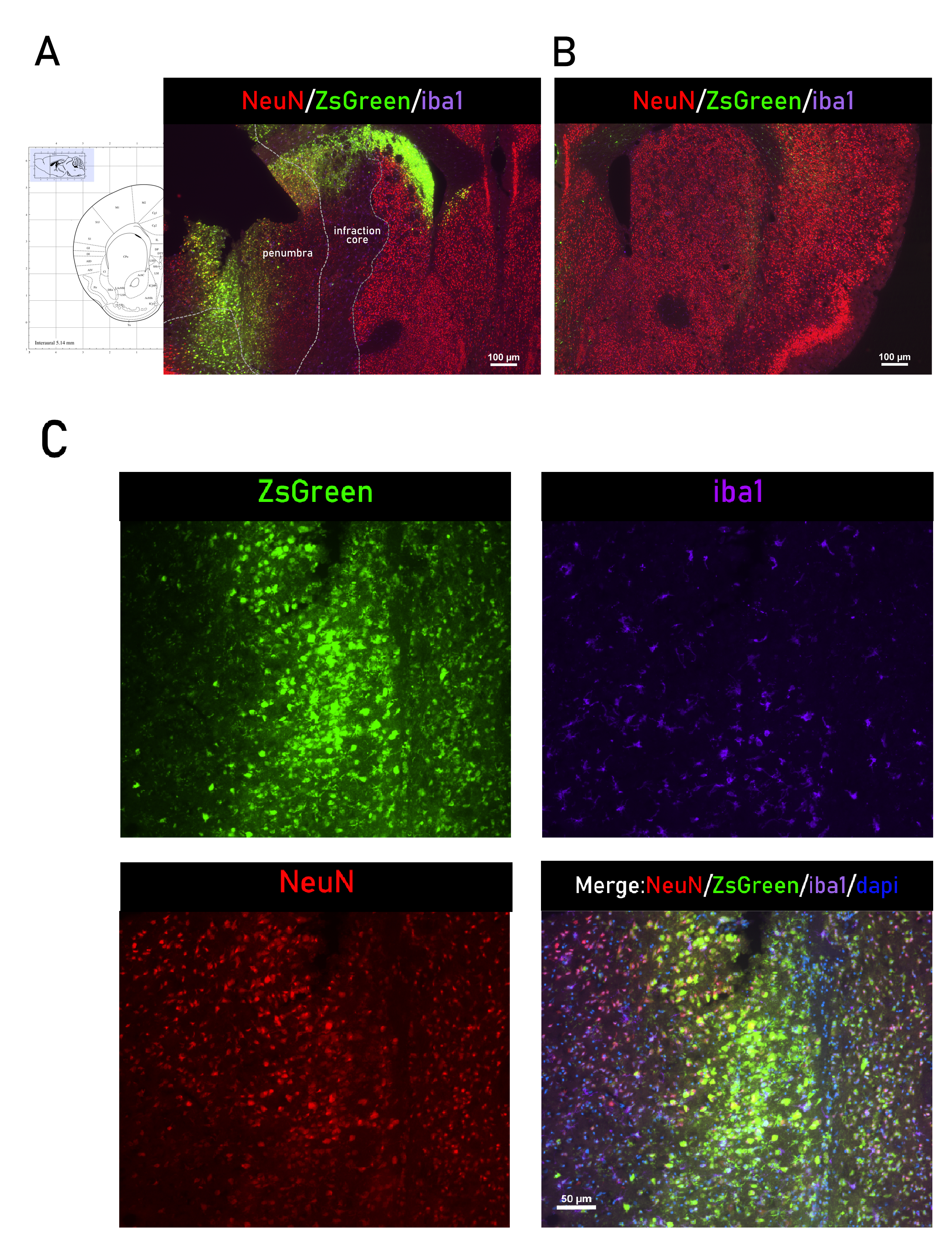

Supplement: Supplementary file 1 — Fig S1 [file CNS-28-566-s004.tif]

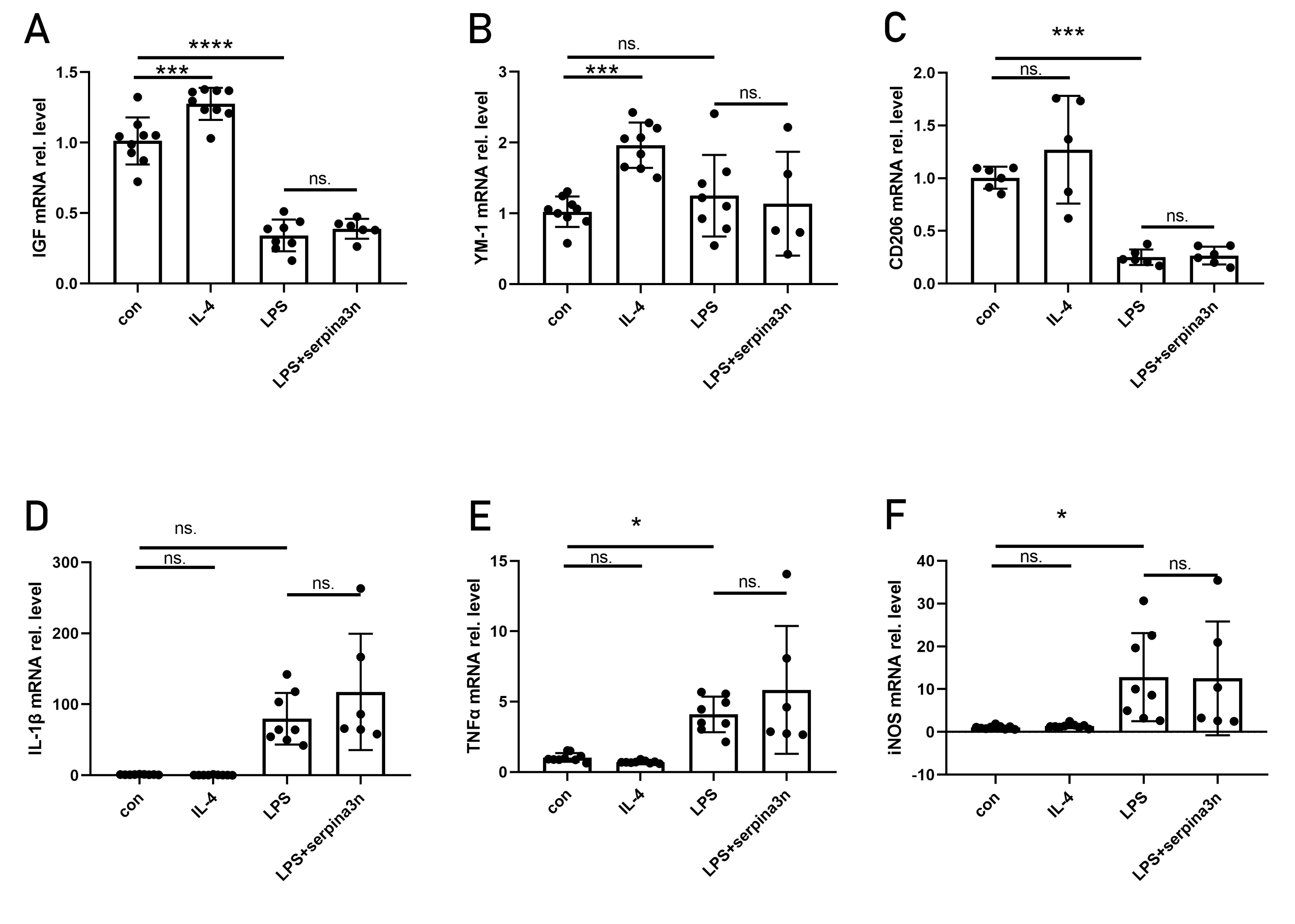

Supplement: Supplementary file 2 — Fig S2 [file CNS-28-566-s006.tif]

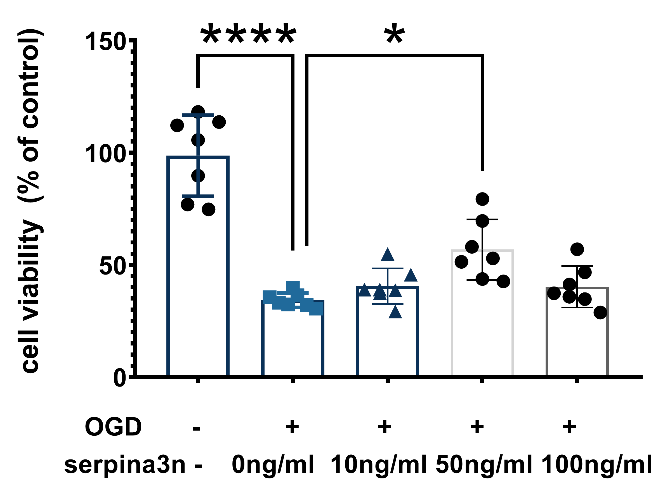

Supplement: Supplementary file 3 — Fig S3 [file CNS-28-566-s005.tif]

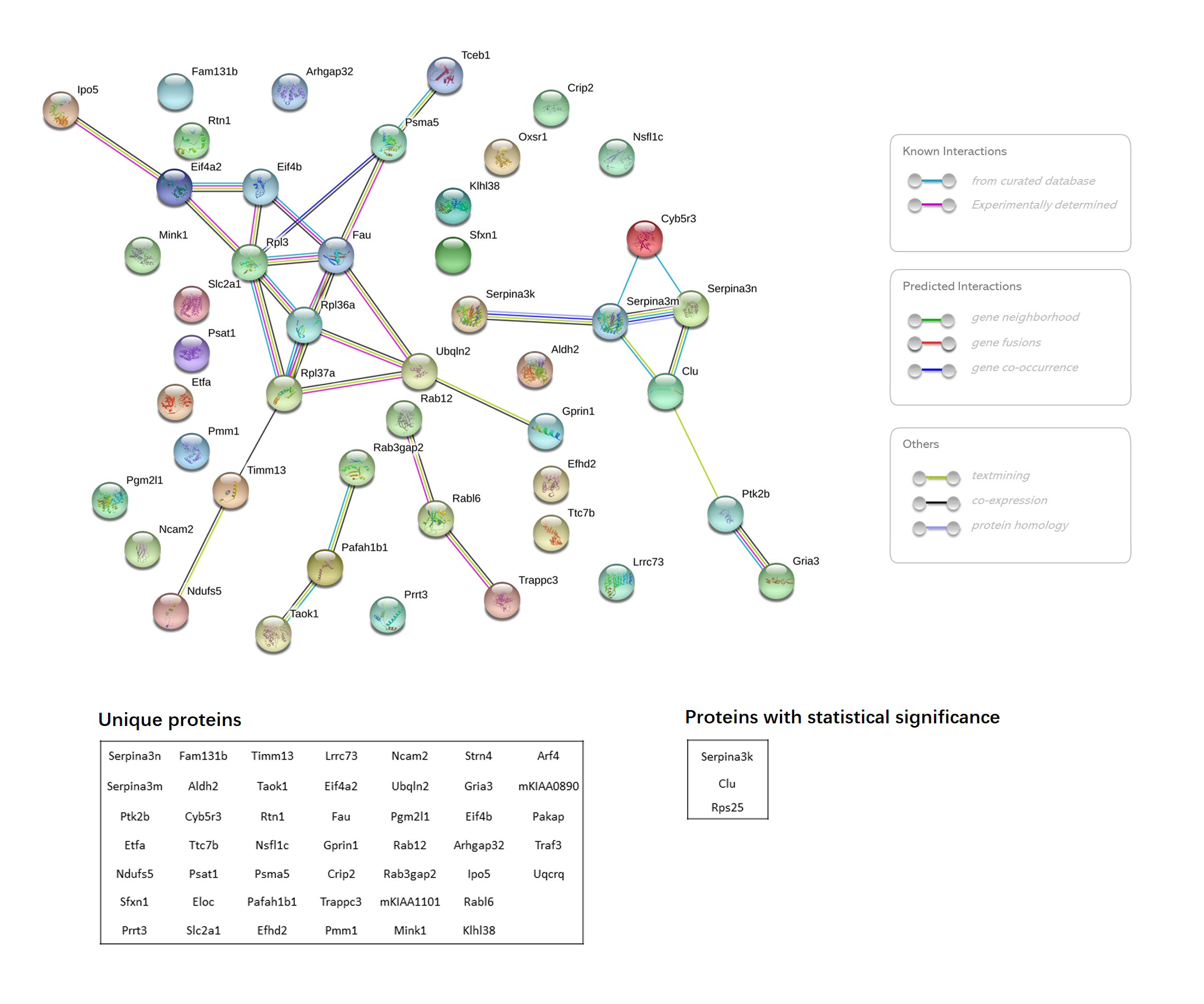

Supplement: Supplementary file 4 — Fig S4 [file CNS-28-566-s002.tif]

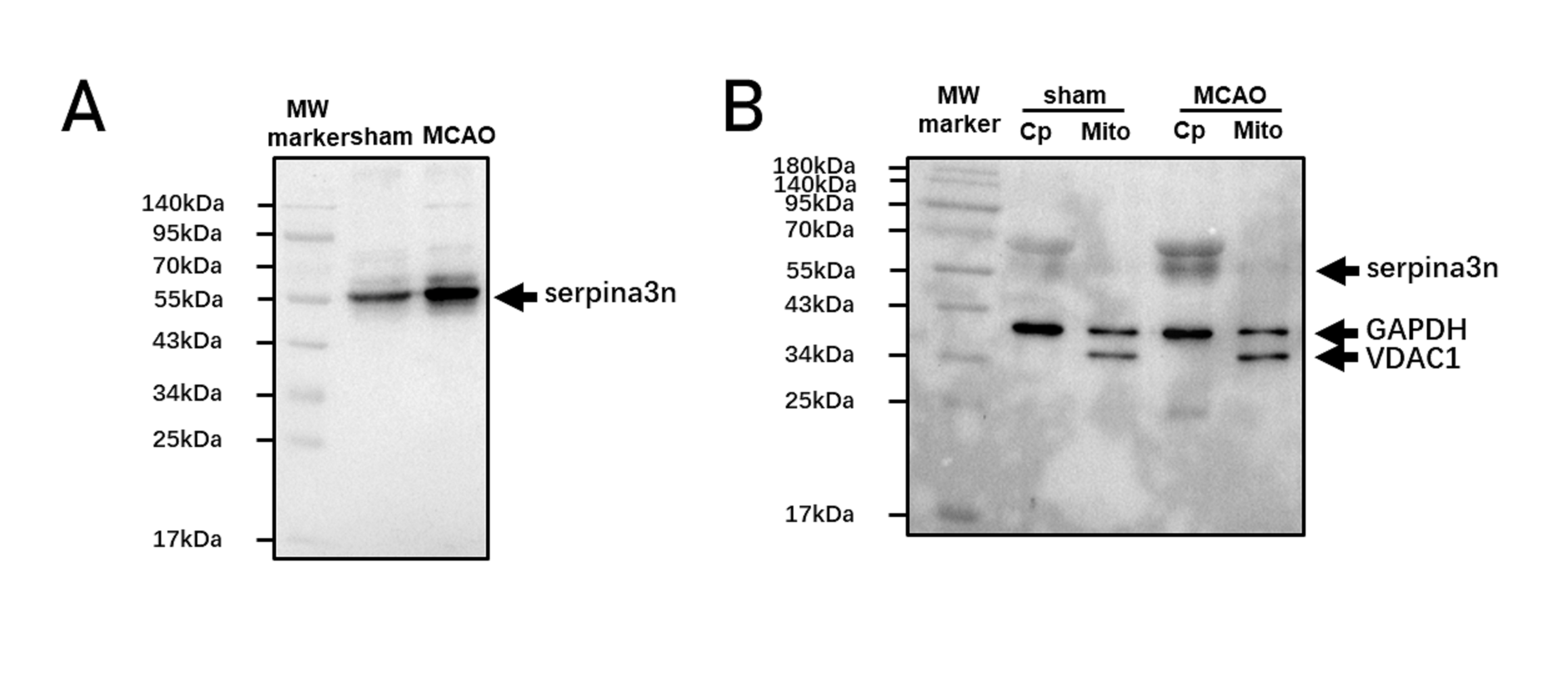

Supplement: Supplementary file 5 — Fig S5 [file CNS-28-566-s003.tif]
