# Supplementary material for: SerpinA3N attenuates ischemic stroke injury by reducing apoptosis and neuroinflammation
Source: CNS Neurosci Ther. 2021 Dec 12;28(4):566–79. doi: 10.1111/cns.13776 (PMC8928918; doi:10.1111/cns.13776)
Supplement: Supplementary file 6 — Supplementary Material [file CNS-28-566-s001.pdf]

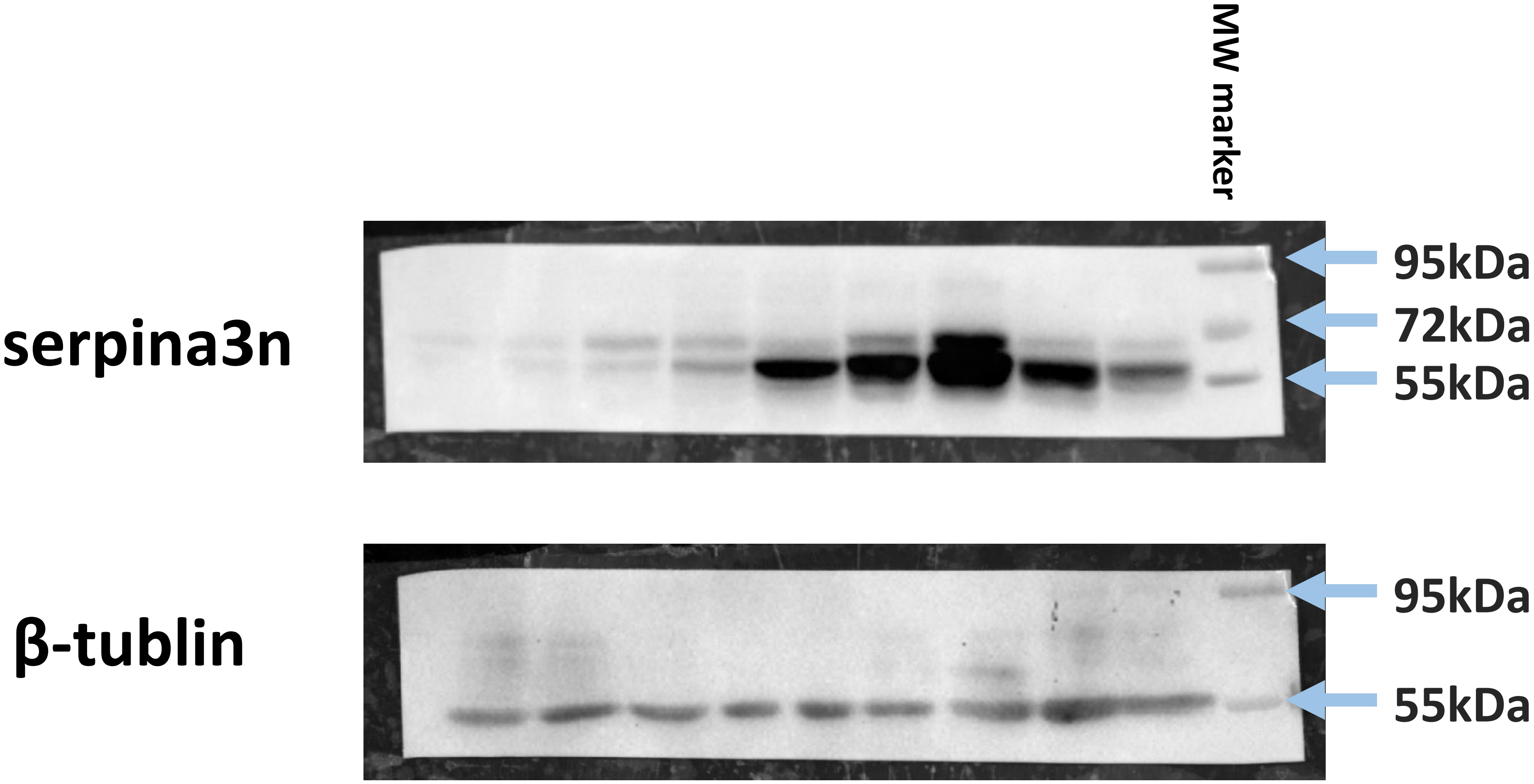

Full unedited gel/blot for Figure 1B in the manuscript

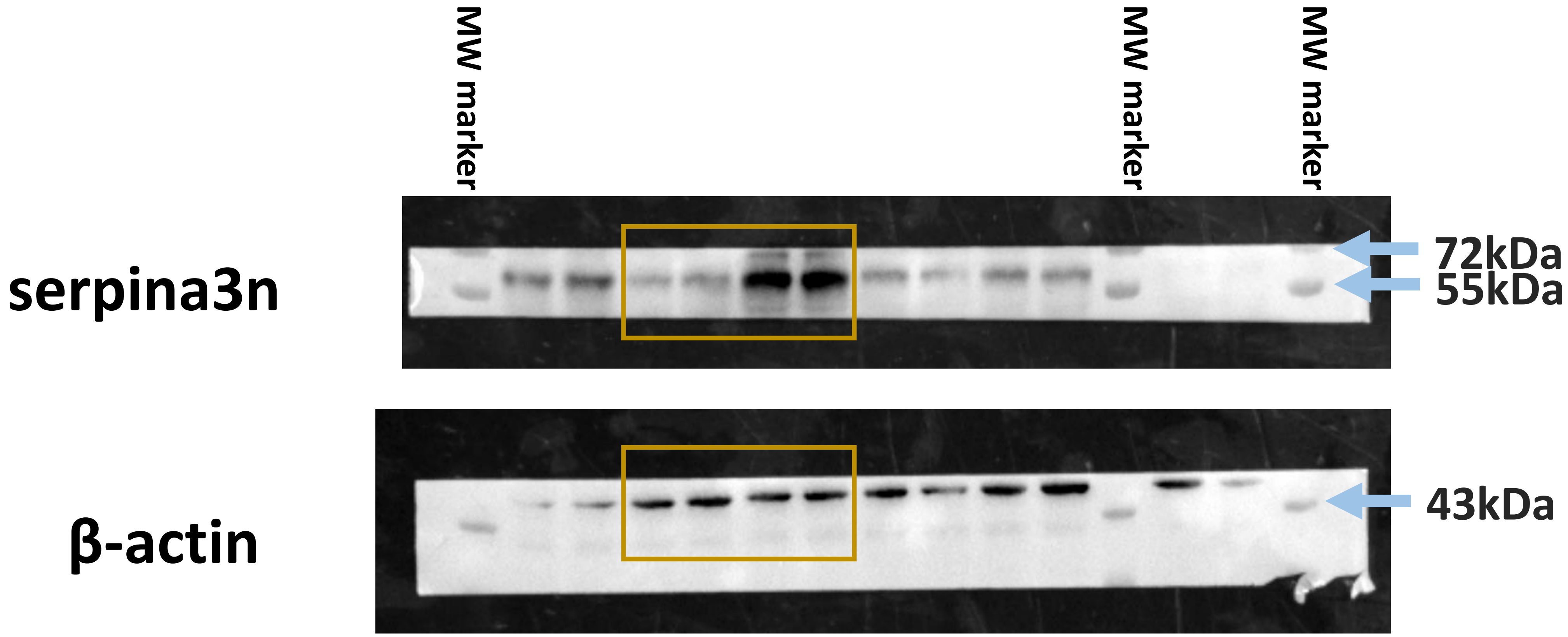

Full unedited gel/blot for Figure 2B in the manuscript

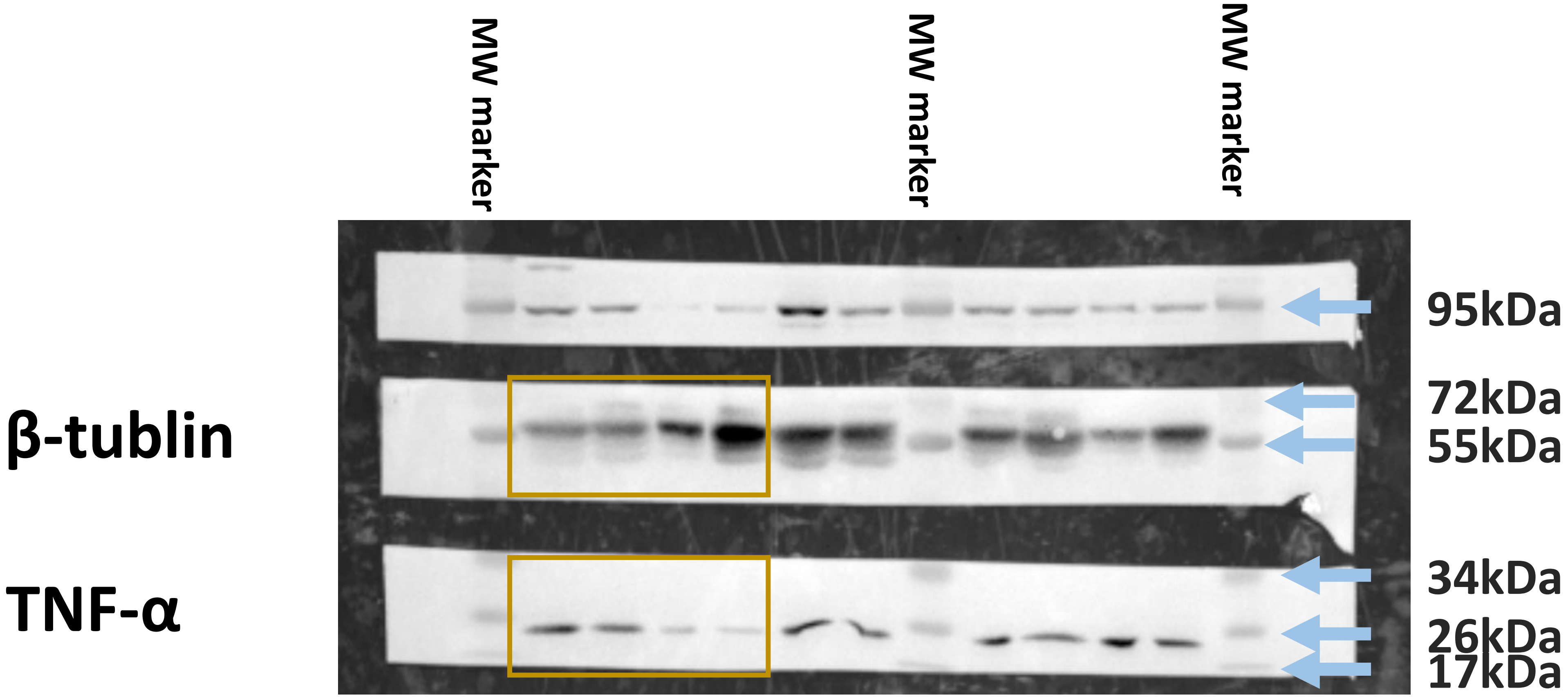

Full unedited gel/blot for figure 3B in the manuscript

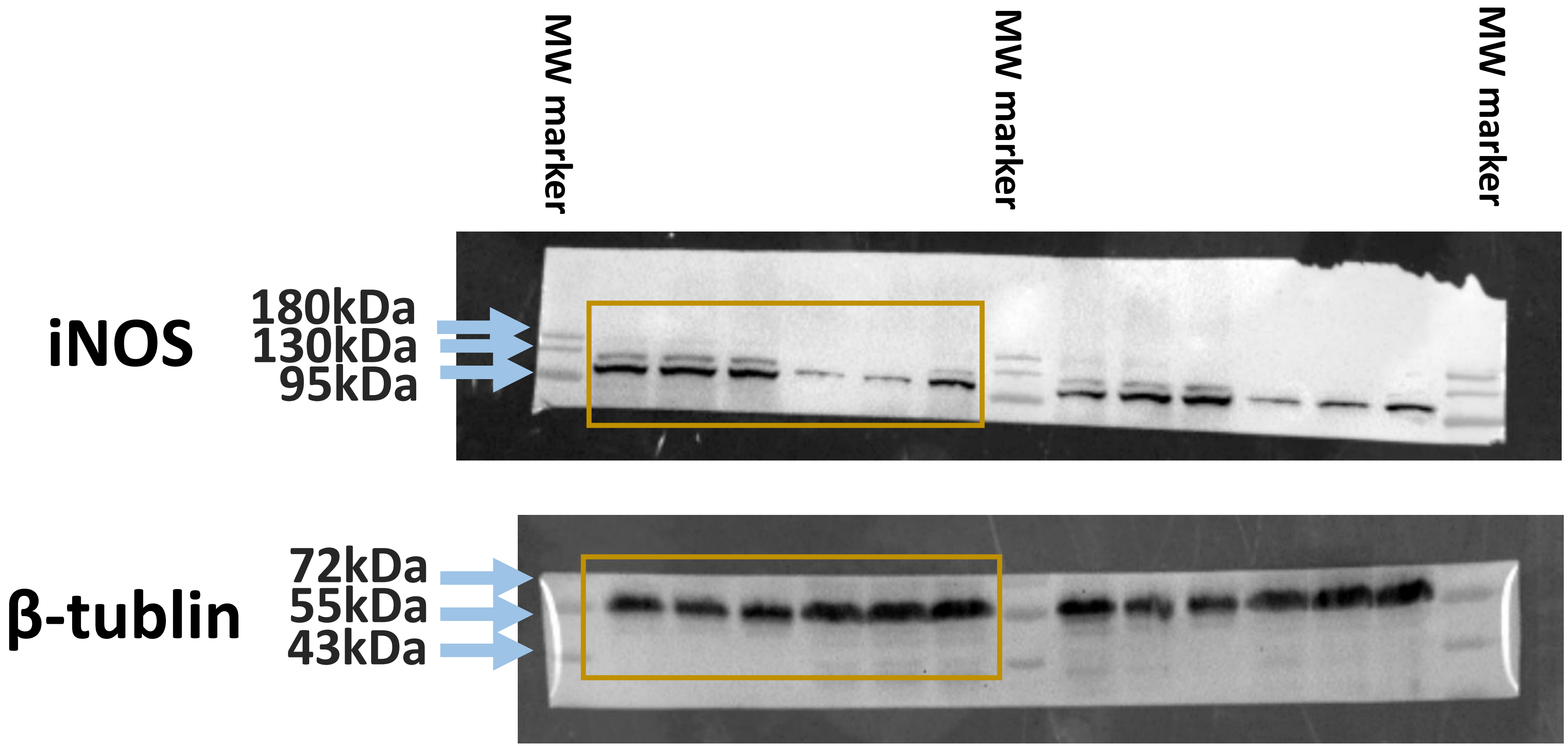

Full unedited gel/blot for figure 3D in the manuscript

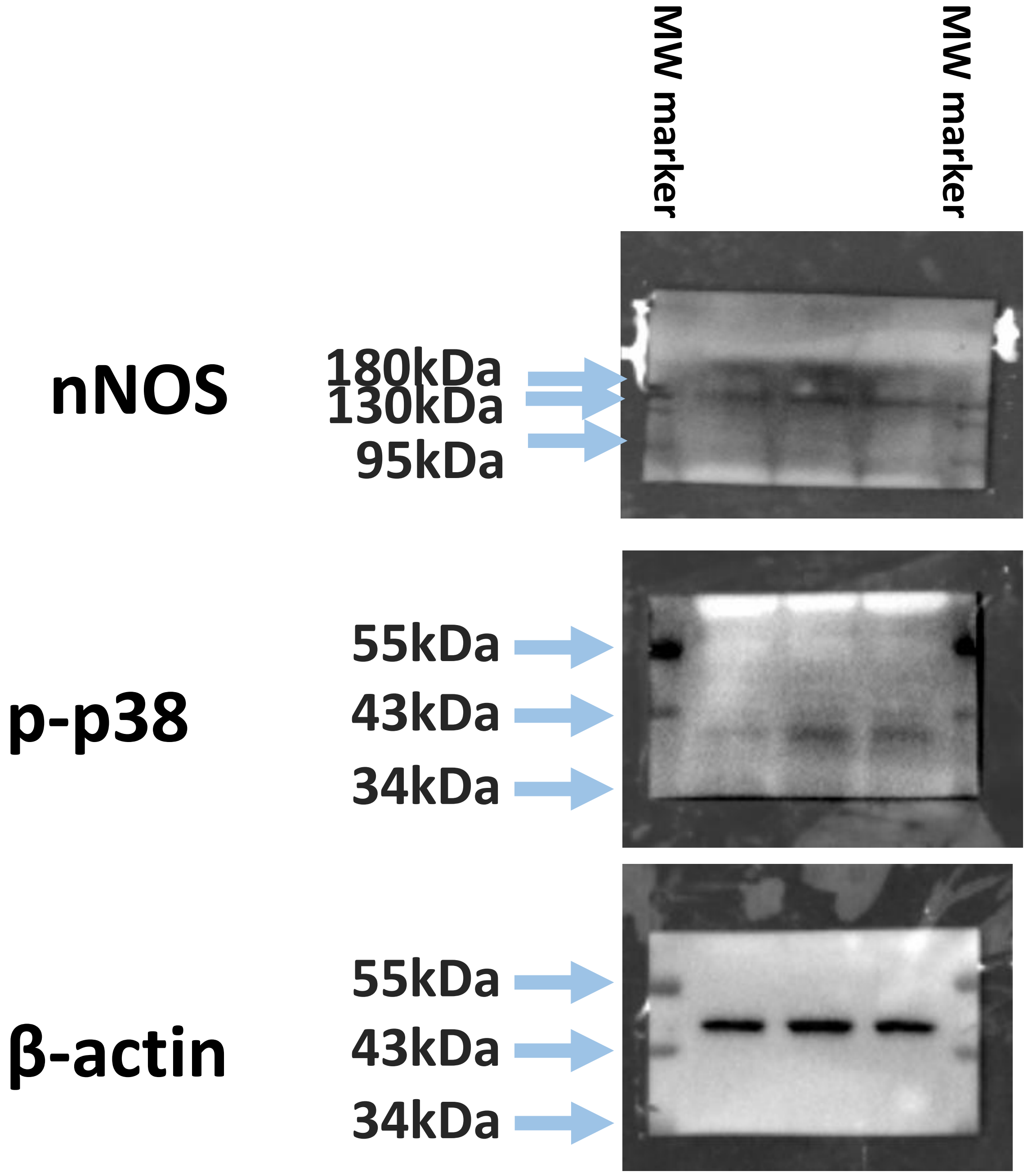

Full unedited gel/blot for figure 4B in the manuscript

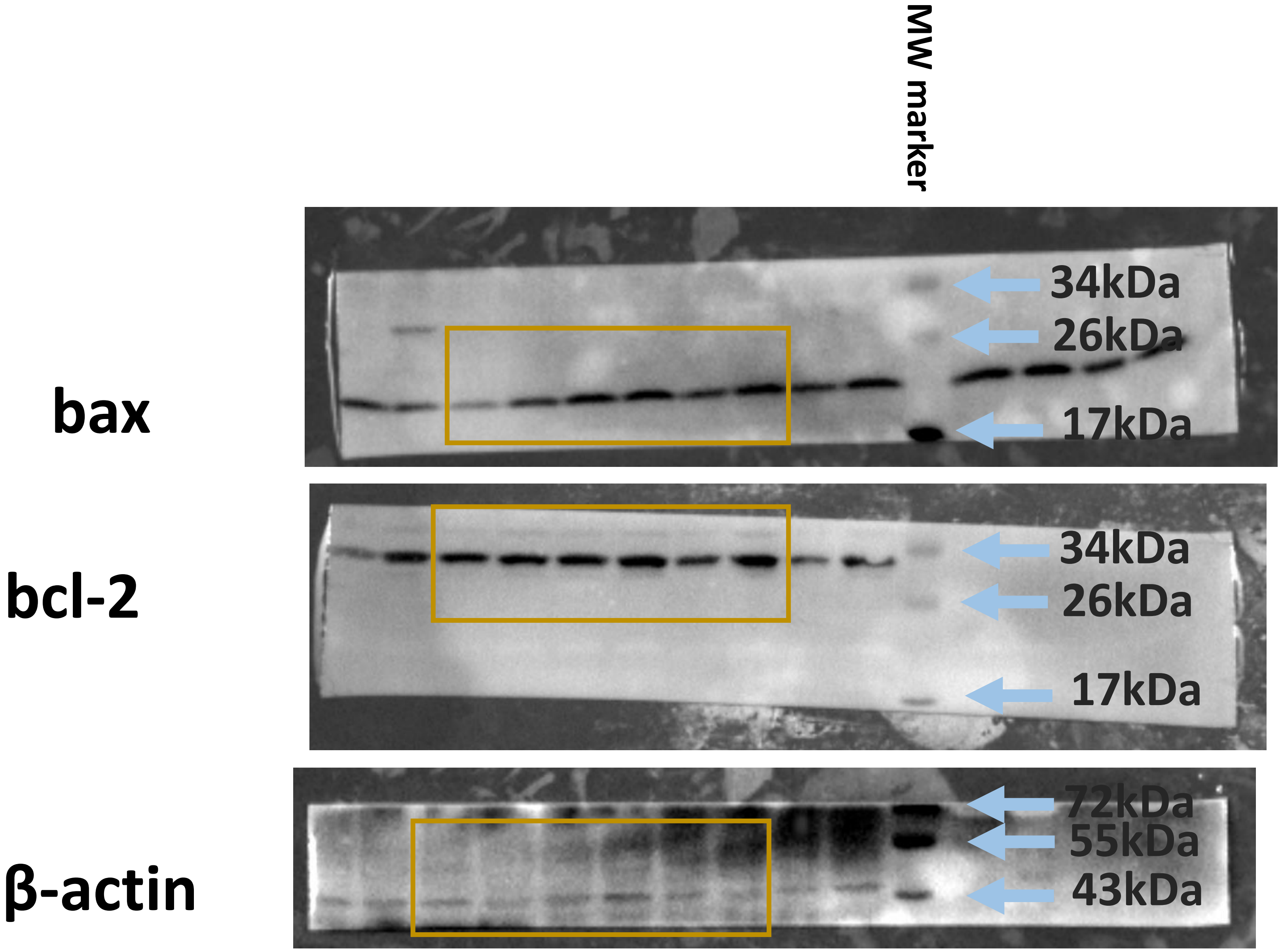

Full unedited gel/blot for Figure 4C in the manuscript

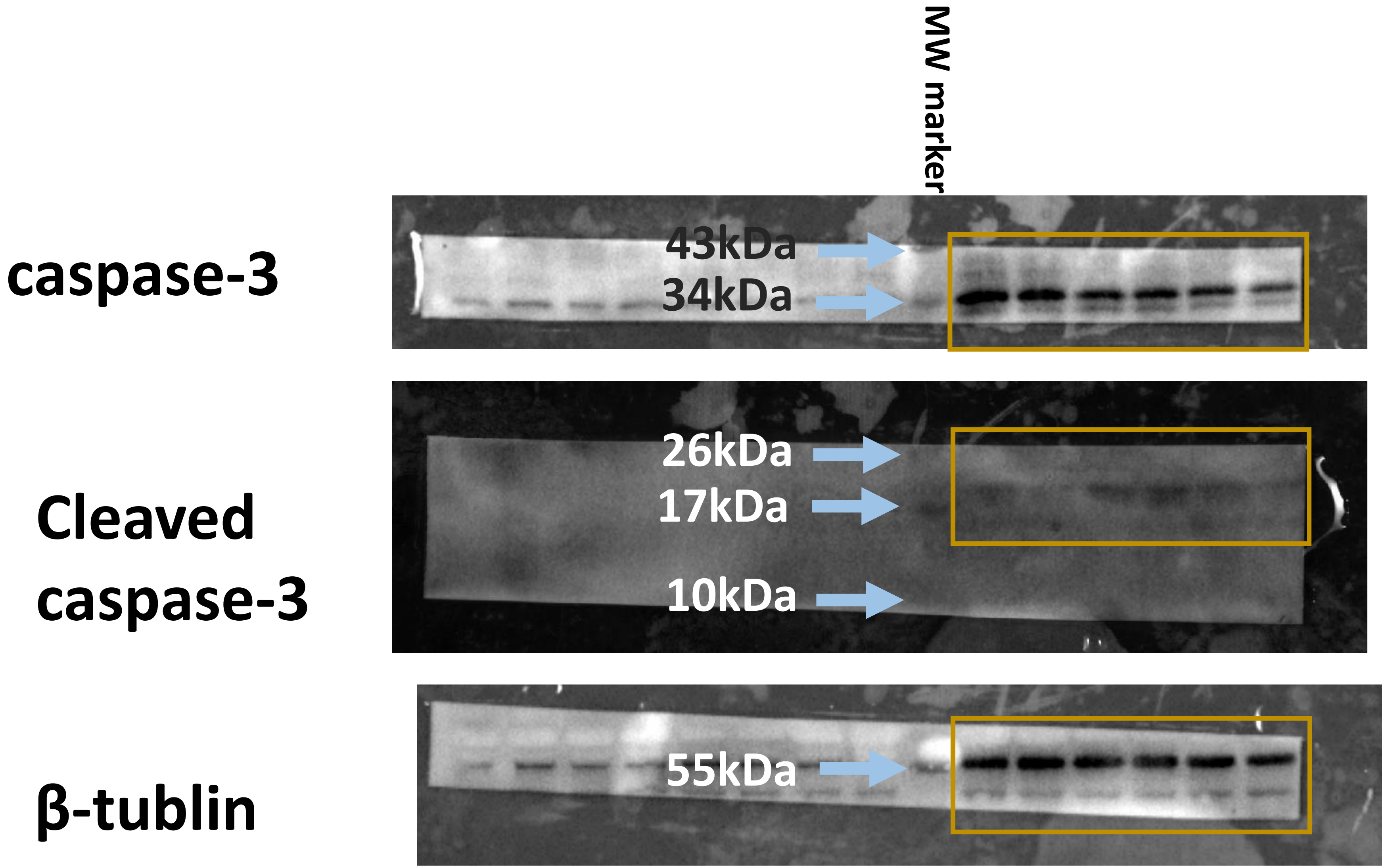

Full unedited gel/blot for Figure 4D in the manuscript

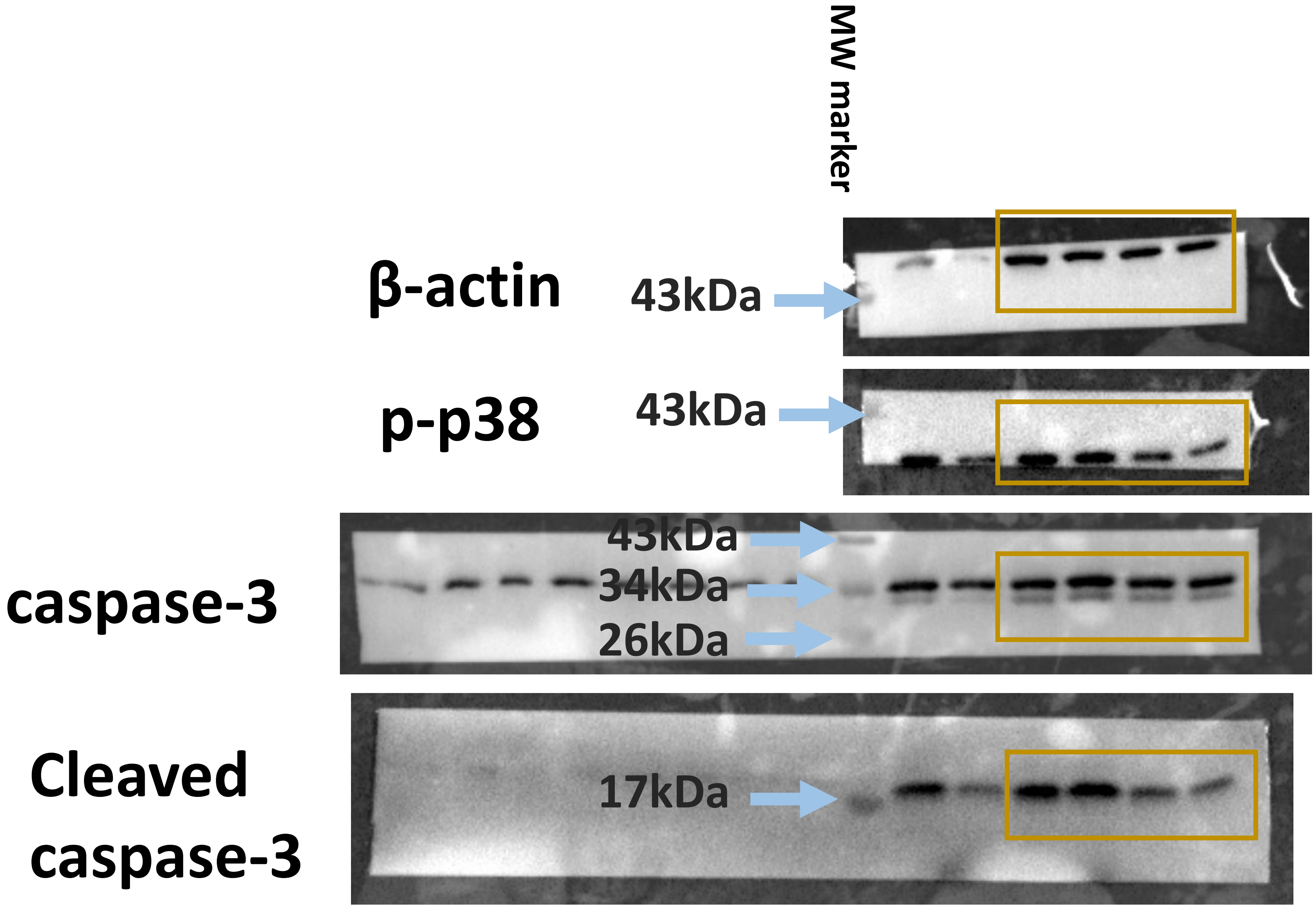

Full unedited gel/blot for Figure 4E in the manuscript

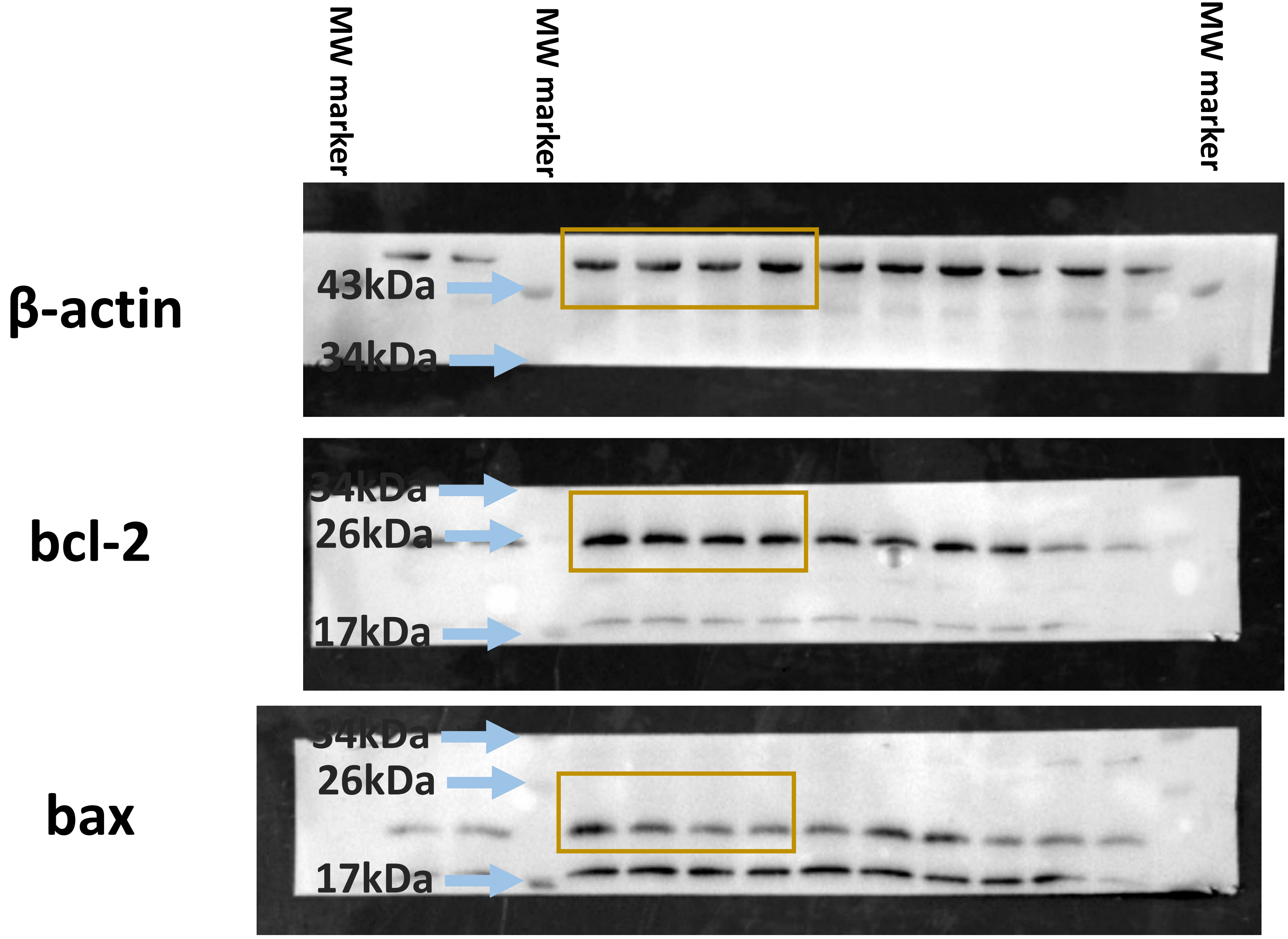

Full unedited gel/blot for Figure 4F in the manuscript

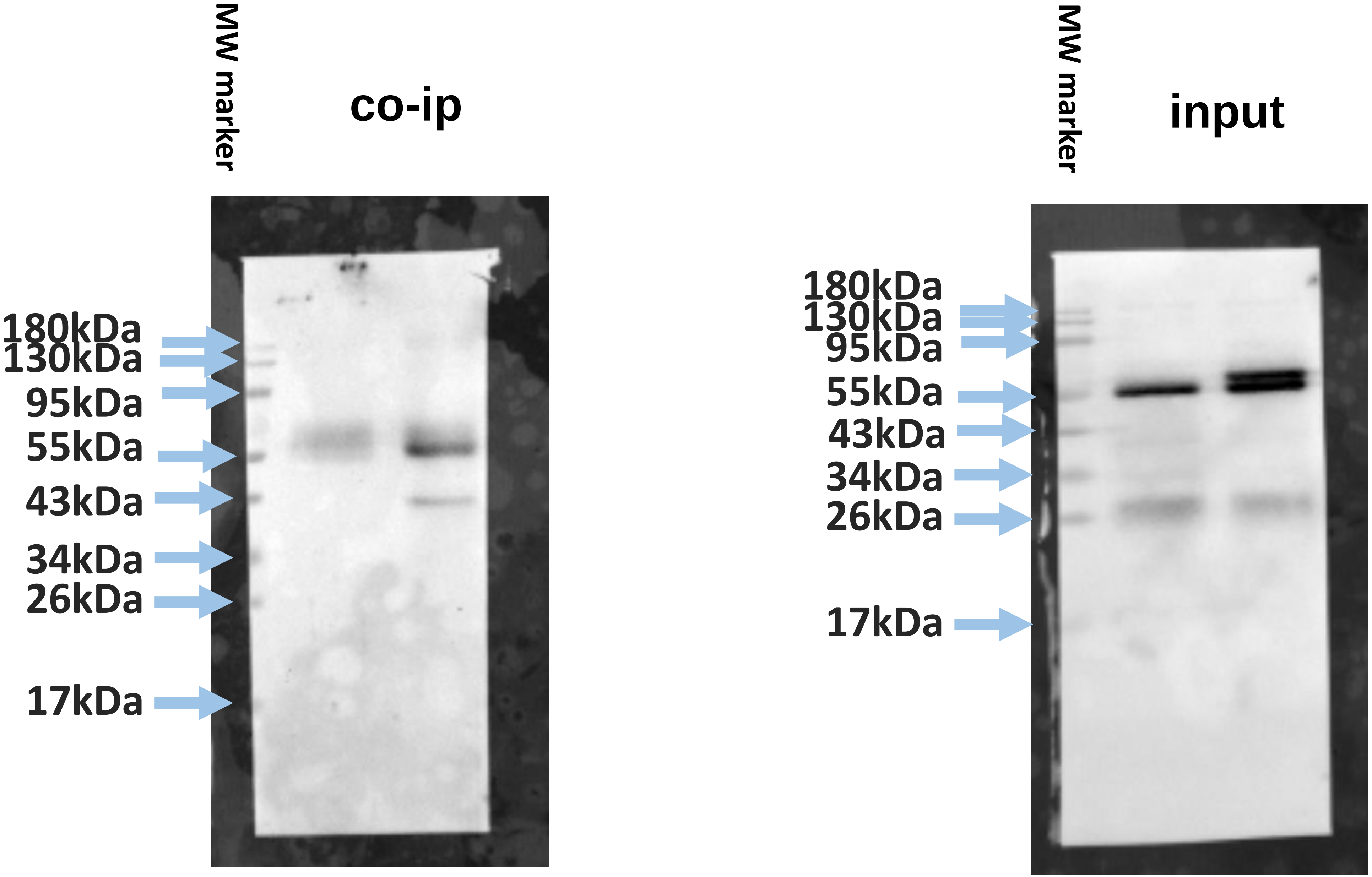

Full unedited gel/blot for F**igure 5A in the manuscript**

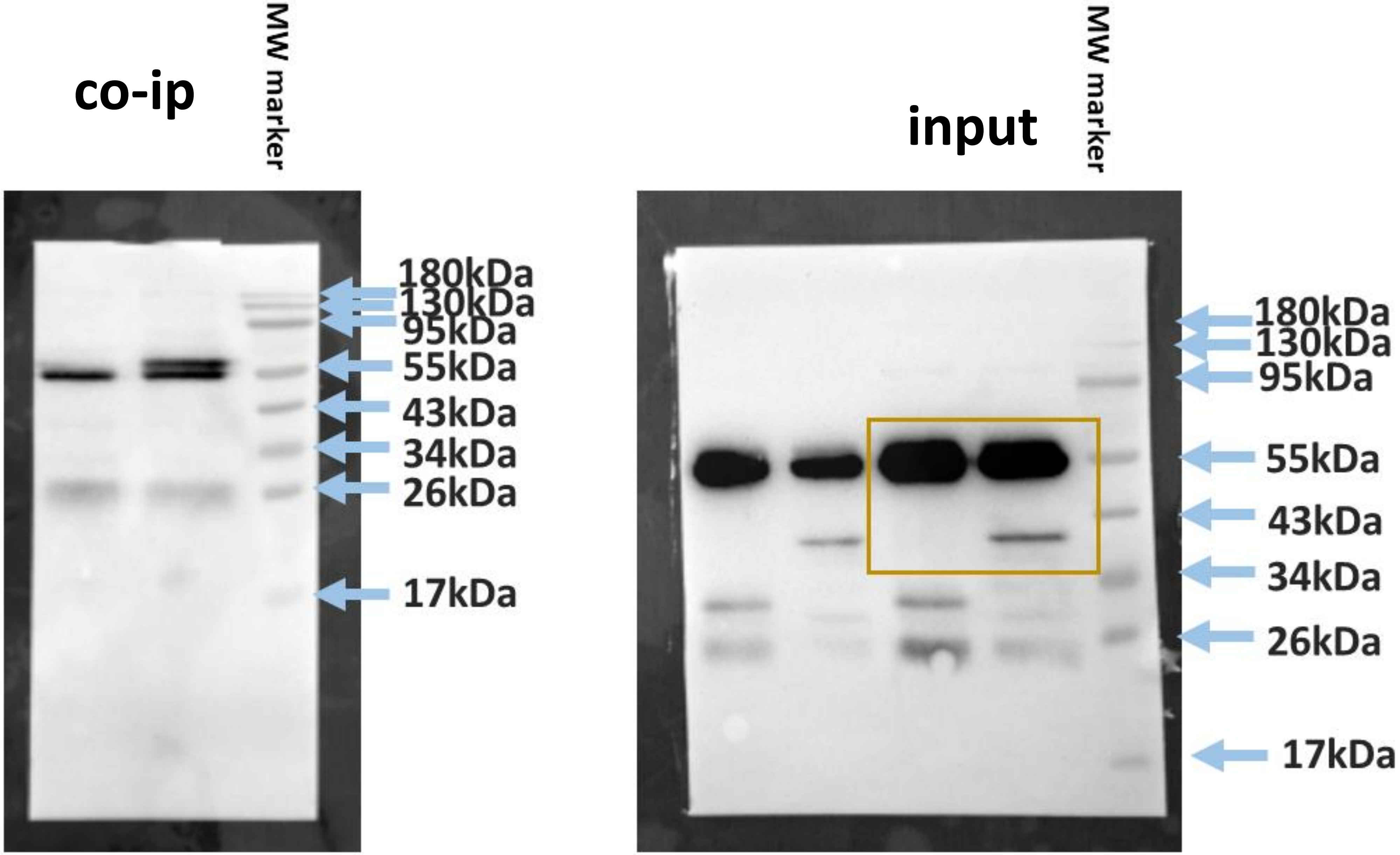

Full unedited gel/blot for Figure 5B in the manuscript

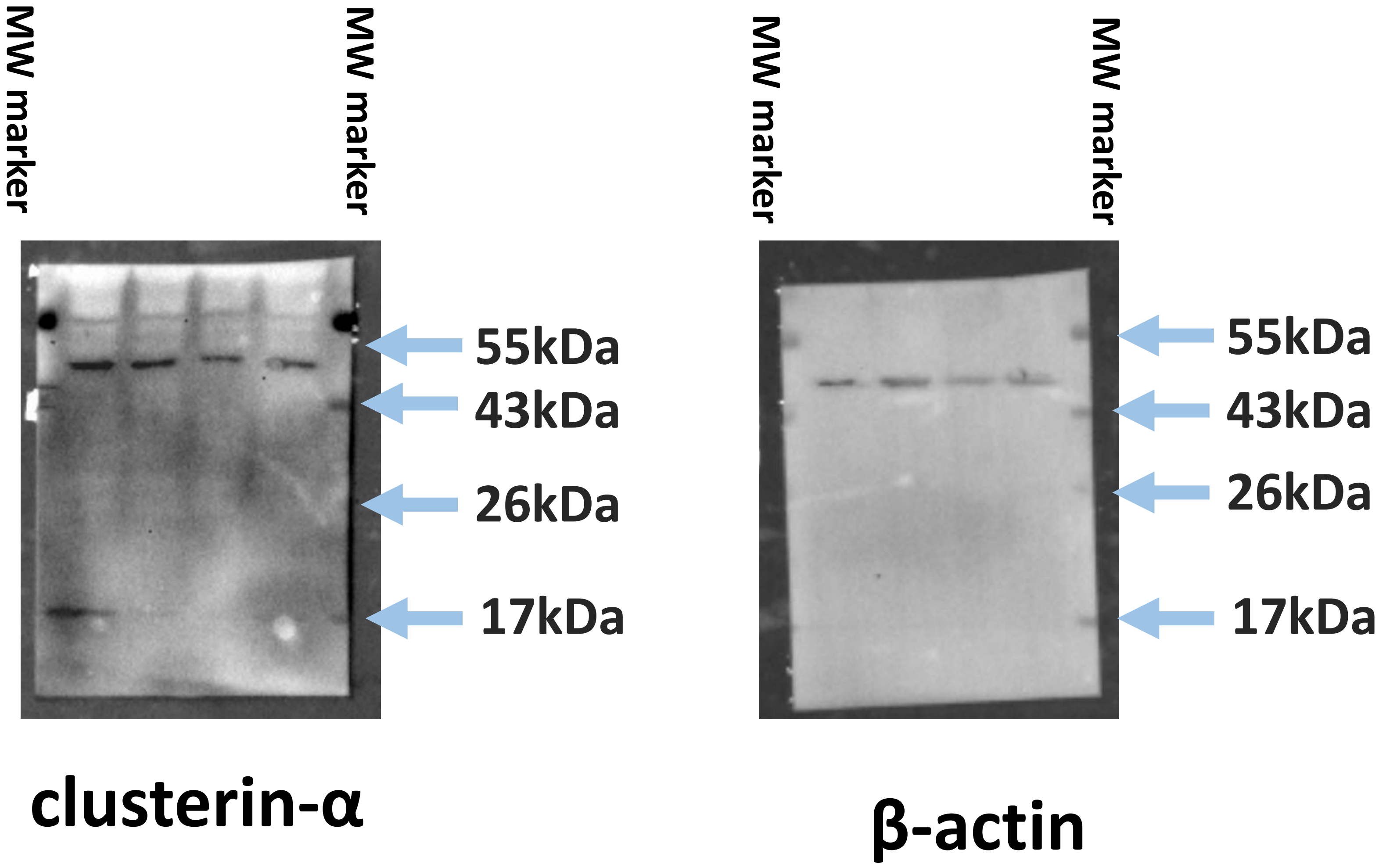

Full unedited gel/blot for Figure 5D in the manuscript

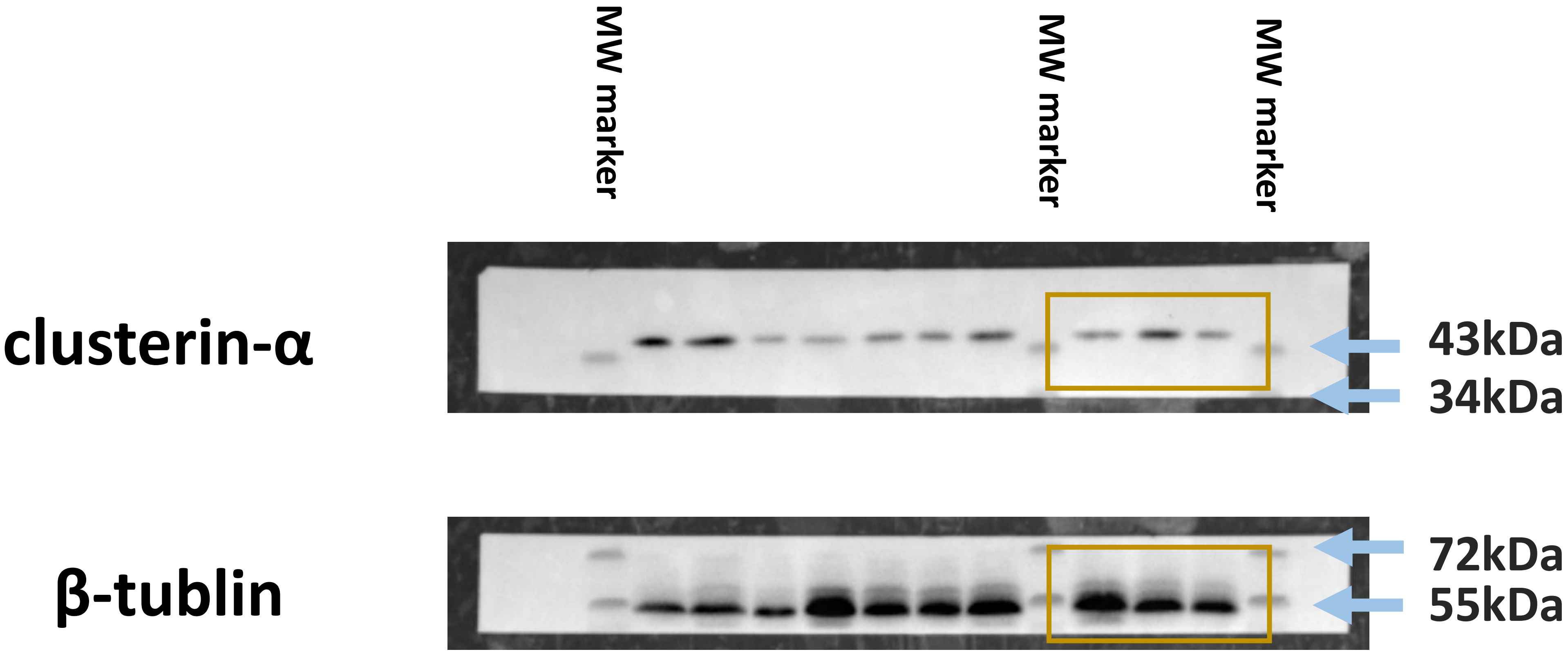

Full unedited gel/blot for Figure 5E in the manuscript

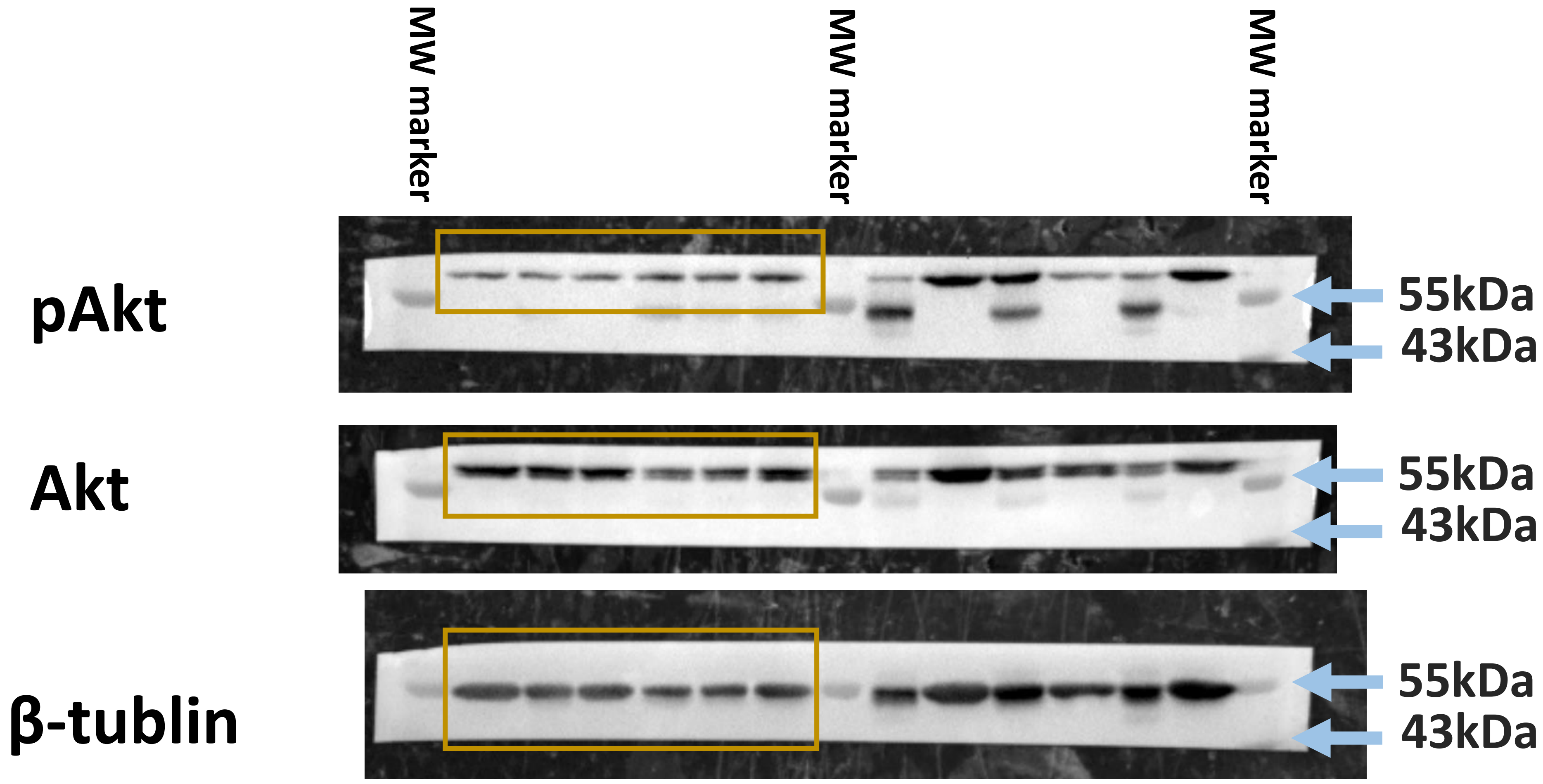

Full unedited gel/blot for Figure 6A in the manuscript

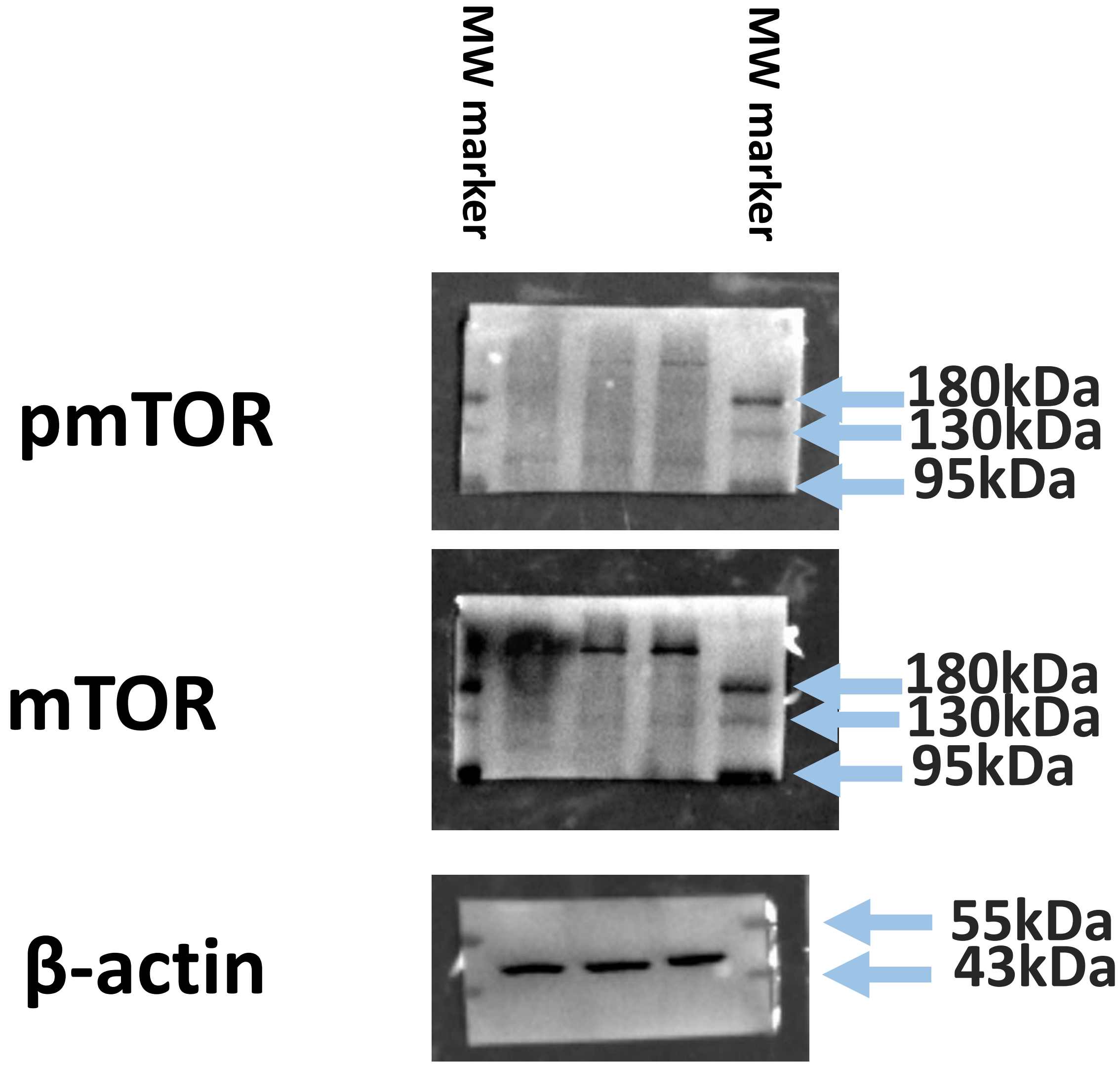

Full unedited gel/blot for Figure 6B in the manuscript

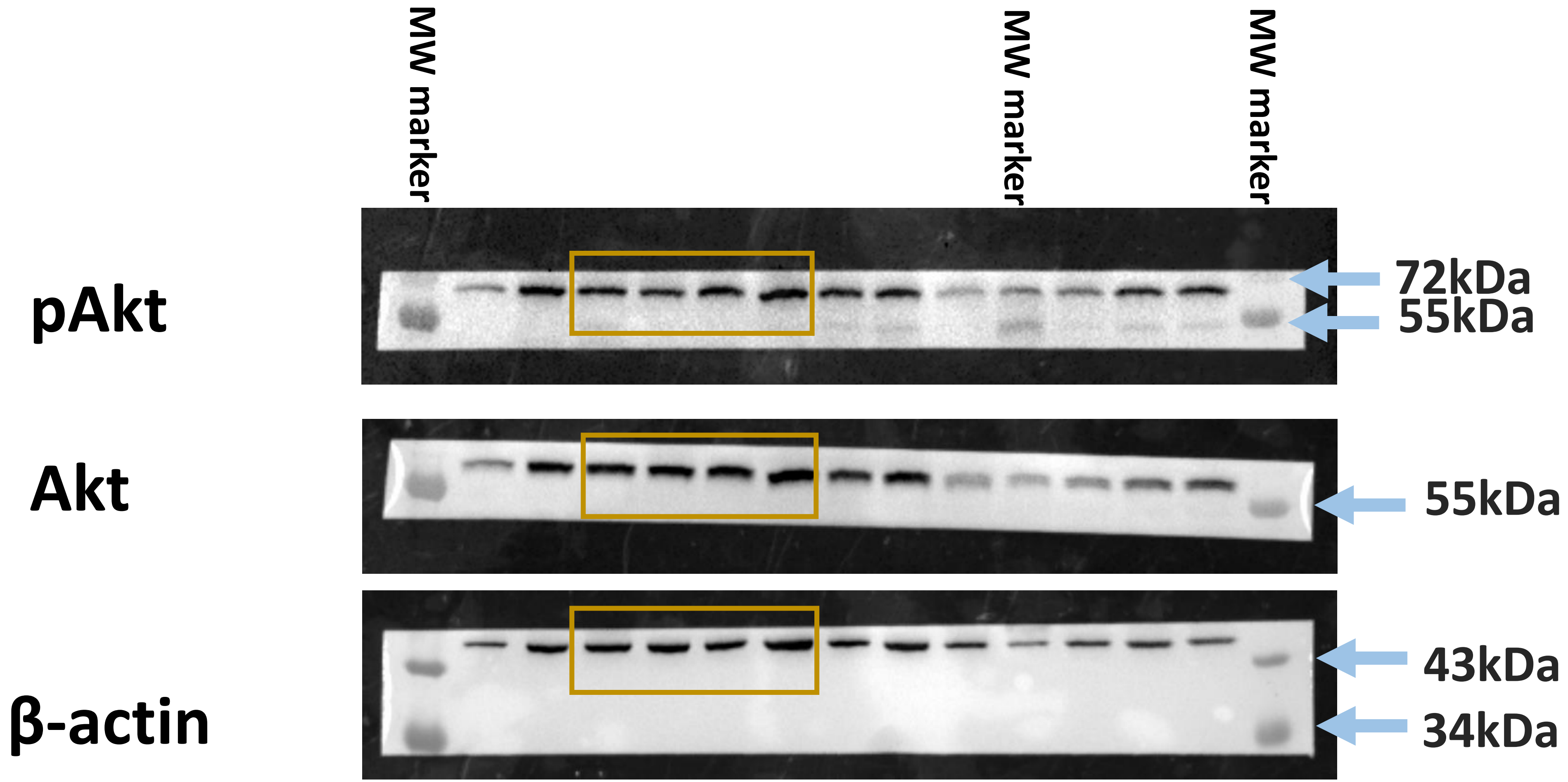

Full unedited gel/blot for Figure 6C in the manuscript

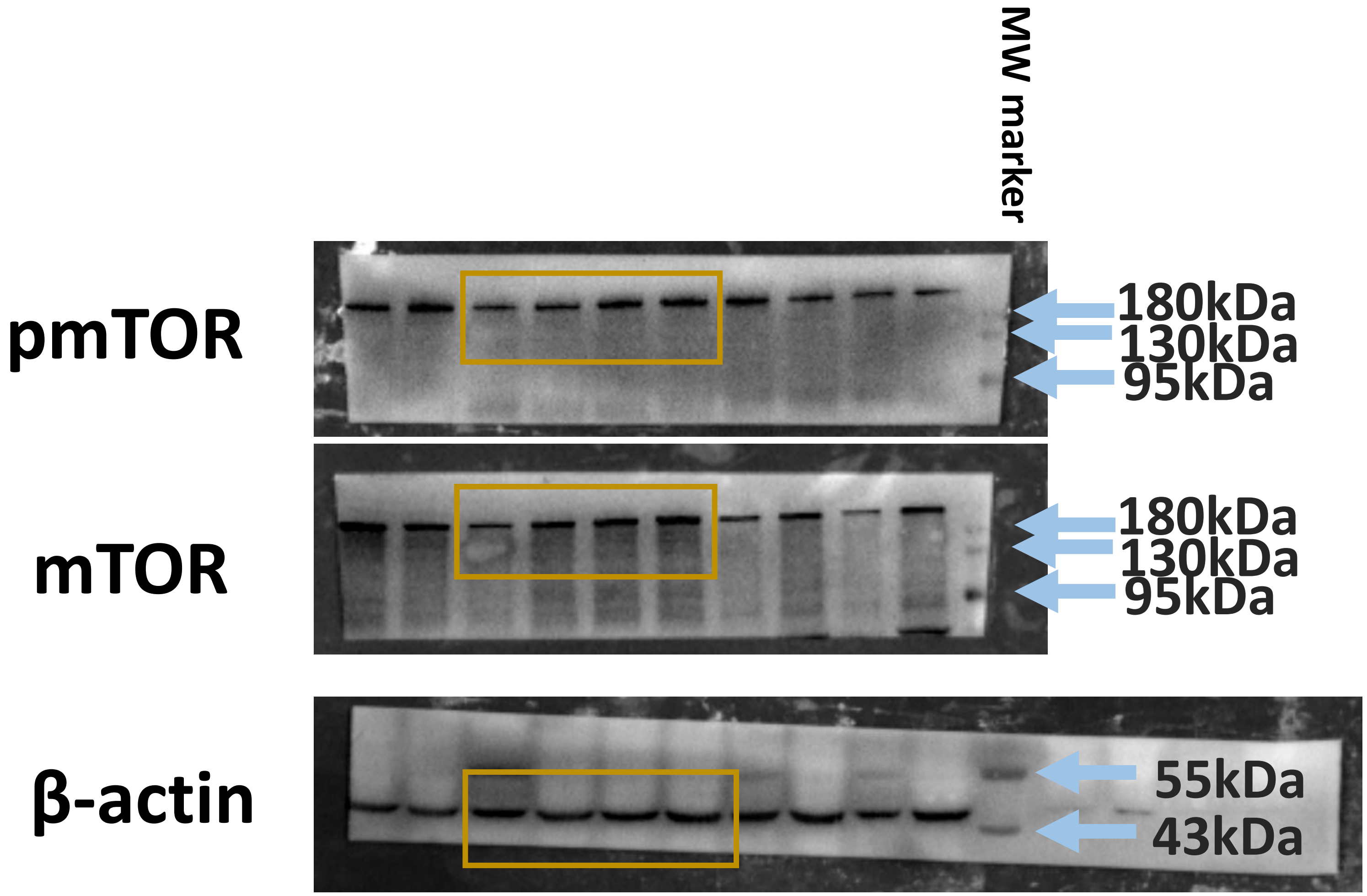

Full unedited gel/blot for Figure 6C in the manuscript
